# Balancing conservation and development in Winter Olympic construction: evidence from a multi-scale ecological suitability assessment

**DOI:** 10.1038/s41598-018-32548-2

**Published:** 2018-09-20

**Authors:** Shuai Song, Sheng Zhang, Tieyu Wang, Jing Meng, Yunqiao Zhou, Hong Zhang

**Affiliations:** 1 State Key Laboratory of Urban and Regional Ecology, Research Center for Eco-Environmental Sciences, Chinese Academy of Sciences, Beijing, 100085 China; 2 School of Environmental & Natural Resources, Renmin University of China, Beijing, 100872 China; 3 University of Chinese Academy of Sciences, Beijing, 100049 China; 4 College of Environmental & Resource Science, Shanxi University, Taiyuan, 030006 China

## Abstract

Optimizing spatial patterns of land development and minimizing the ecological impact of concentrated construction is the key to realizing regionally sustainable development. The reasonable assessment of the ecological effects of the Winter Olympic construction on areas where the mountainous ecosystem is ecologically sensitive and vulnerable is urgent for responsible urban and regional development. Here, we assess the multi-scale suitability of ecologically compatible development in Winter Olympic regions using the ecological suitability assessment method based on GIS spatial analysis. We found the Chongli District had relatively high ecological structure and function resistances at a basin scale and that the towns where Olympic facilities located also had larger ecological resistance. The integrated suitability assessment showed the prior and moderate zones for suitable large-scale development and utilization in Chongli were smaller than those in other counties. The total loss area of natural ecological systems (forests, shrubs and meadows) for a new ski resort is 117.27 hm^2^, which will lead to ecosystem function loss such as water and soil conservation and will potentially impact ecological systems. This research will be a useful reference for exploring the multi-scale balancing of conservation and development for Winter Olympic regions, and in turn, for concentrated global constructions.

## Introduction

The relationship between regional development and ecosystem conservation has been the subject of decades of intensive research^1–3^, especially in some national strategic planning construction areas^4^, e.g., Olympic construction^5,6^. The regional development accompanied by land use structure changes is dependent on both territorial characteristics and land use policies^7,8^. The analysis of ecological suitability driven by historical, ecological and socioeconomic analysis may contribute to sustainable land management principles^9,10^ and the sustainable development of urban and rural areas^6^. Beijing and the neighboring city Zhangjiakou in Hebei province have announced a joint bid to host the 2022 Winter Olympics, in which events staged on ice will be in Beijing, and the snow events will be in Zhangjiakou. Concerns have been raised regarding the potential ecological impact of the 2022 Winter Olympics on surrounding regions^11,12^. The Winter Olympics could not only affect the current landscape pattern but also drive the distribution of regional investment, population layout and redistributed land use patterns in the future. Each part of planning the construction for these events or future land use management represents a significant source of anthropogenic disturbance, while also providing recreation and revenue^13^. Among the available landscape modifications, some are particularly high-risk and are likely to exert a negative influence on the ecosystem structure and function^2,14^. Generally, high-risk situations include a vulnerable natural ecosystem, the presence of sensitive species, proximity to wetlands and water ecosystems, the presence of cultural or historical value requiring strict protection or sites that have been the subject of public protest leading up to the Games. Regional land use management including the current ski areas is a conservation concern because they significantly alter fragile high-elevation ecosystems^15,16^. Destruction to natural ecosystems and metabolic imbalances greatly threatens regional ecological security^13,17–19^. Therefore, to ensure effective ecological constraints on development and construction, it is essential to conduct an ecological suitability assessment.

Currently, the ecological suitability evaluation for land construction focuses on the paradox between ecological protection and construction on land for rapid expansion^20,21^. This method is able to identify the location and ecological boundaries of suitable areas for construction based on grading index factors through GIS-based rule settings such as cellular automata^22^, fuzzy measures^23,24^, analytical network process^25^, urban growth model^26^ and spatial clustering^10^. Overall, the basic principles of existing studies are similar to those utilized in this study: optimizing the adaptive hierarchy through remote sensing and GIS spatial analysis techniques^20,21,27,28^. This approach has also been extensively applied in the assessment of different kinds of land use types, such as agricultural land^29^, land habitats for animal and plant species^30^, landscape evaluation and planning^31^, ecosystem services sustainability^32,33^, and environmental impact assessments^34^. For example, the regression neural network method and ecological suitability conceptual model were used to evaluate the land suitability^10,35^. Additionally, the ecological suitability theories have been expanded by studies examining landscape ecological security patterns and ecosystem services^36,37^ and dynamic ecological processing frameworks^10,38^. However, most of these ecological constraints add ecological factors to the model^10^ rather than investigate the relative supporting roles, essentially neglecting the effects of simulation in different scales and the evaluation of ecological loss of construction through ecological field survey.

This paper aims to assess the suitability of ecologically compatible development in Winter Olympic regions by using multi-scale GIS spatial analysis methods. The spatial scales of the study area includes the Yanghe watershed, Chongli District, and the Olympic ski area and ski piste. More particularly, we give priority to the future of independent development in the Yanghe basin, while focusing on risk management and trade-off analysis in the Chongli District. Meanwhile, through evaluating the loss of ecological benefits, we stress the need for better ecological protection and compensation in ski resorts and their areas. To reduce the negative impacts of human activities, to decrease ecological risks, and to add to the quality and fun of the Games in construction areas, this paper examines the spatial dynamic characteristics of a natural ecosystem’s self-organization and capacity to self-update, reflected in terms of the three aspects of ecological elements, ecological importance, and ecological resilience by using the integrated ecological resistance (IER) conceptual model based on ecological circulation theory.

## Results

### Ecological structure-function-dynamics resistance for the Yanghe watershed development

**Figure 1:**
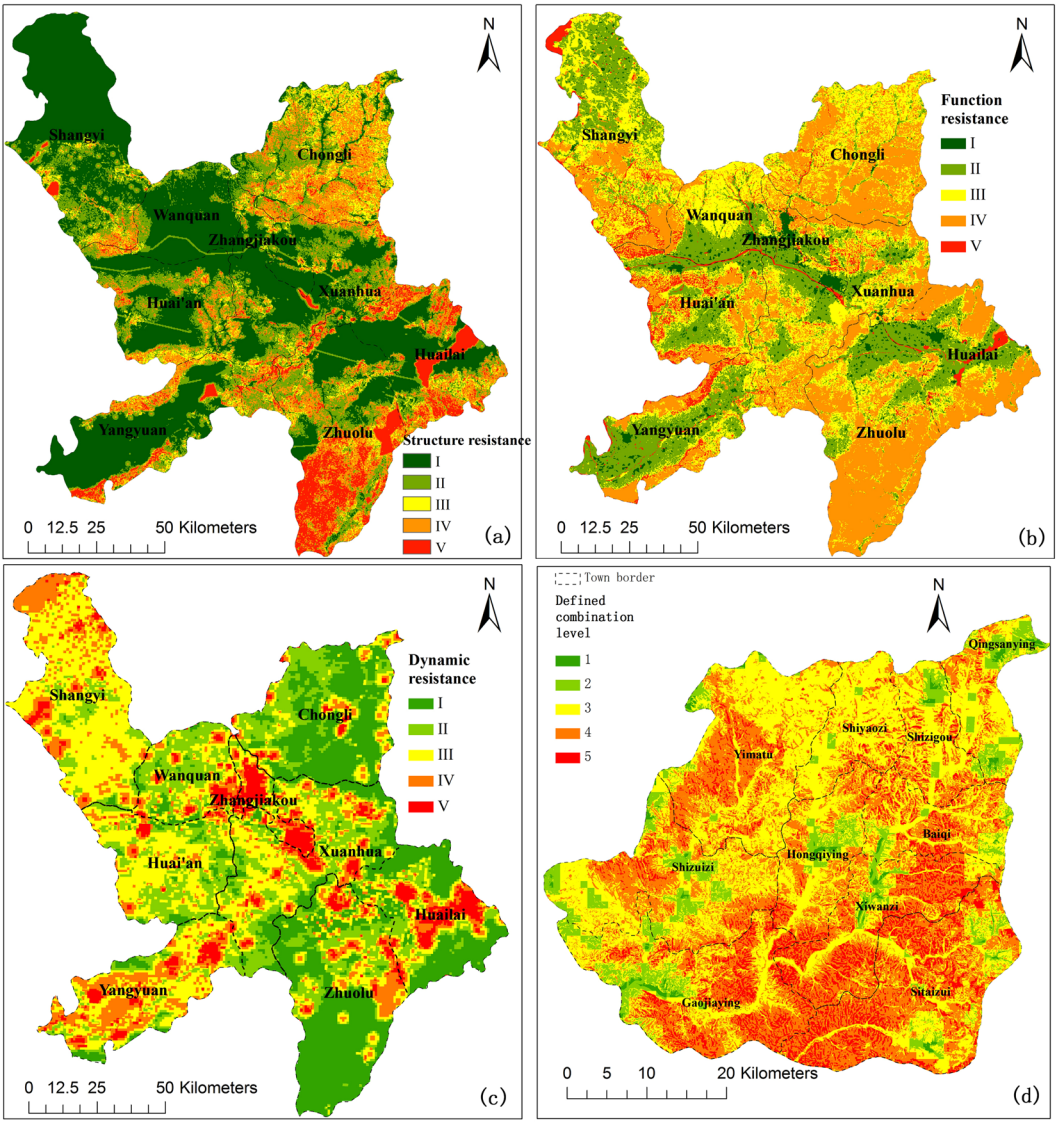
Spatial distribution of ecological structure resistance (**a**), ecological function resistance (**b**) and ecological dynamics resistance (**c**) for basin development. (**d**) Spatial patters of combination of individual resistances in Chongli County. Level 1: three-low; level 2: two-low; level 3: three middle or two middle; level 4: two-high; level 5: three-high. The figure (**a**–**d**) is generated by ArcGIS 10.1 software, http://www.esri.com.

Spatial distribution of ecological structure resistance (Fig. 1a) determined by topography, geological environment, and ecological protection zones shows that the Yanghe watershed areas are generally at lower levels, with the first and second category together accounting for 66.79%, and indicating most areas are suitable for land development from an ecological structure perspective. Results also showed that the more stable geological conditions and the more favorable elevation and slope conditions, the ecological structure resistance will be lower (Figs 1a and S1–S3). The highest (7.95%) and the fourth resistance category (19.01%) account for a relatively small proportion, which is mainly distributed in mountainous areas. In comparison with structure resistance in the west area, the east area was obviously higher. For instance, Huailai County is a basin with mountains in the north and south and the Guanting Reservoir (natural conservation area) in the center, accompanied by relatively high ecological structure resistance (the fourth and fifth categories together, 43.25%). Chongli District, as the focus construction area for the Winter Olympic venue, has a relatively high ecological structure resistance, where 43.93% of the area belongs to the higher grade (fourth and fifth level) and only 9.16% of the area belongs to the lowest category (Table S1). On the other hand, although higher elevations and steeper slopes are not suitable for development and construction on a regional scale, slope conditions of 15°~35° are more conducive to the establishment of a skiing track.

The spatial pattern of ecological function resistance is revealed by the integrated characterization of water-soil conservation and biodiversity protection (Figs 1b and S4–S6). The surface water in the watershed is extremely scarce, and the Guanting Reservoir, an important backup water source, is facing great pressure from upstream pollution^39^. To protect this water source, which is an important supplier of water, and to support the regional ecosystem function, ecological function resistance of land-cover data containing water bodies is directly defined at the highest level. The proportions of the total area for the highest resistance category and lowest resistance category are very small, 5.09% and 3.15%, respectively, while the moderate level and fourth level together account for 64.86% of the total area (Table S2). The ecological function resistance of Zhangjiakou was remarkably low, which reflects the continuous disturbance of human activities on the surface cover in a rapidly urbanizing city. By contrast, Zhuolu and Chongli (fourth and fifth function resistances, 61.79% and 58.45%) are mountainous counties with high-density vegetation cover, where the mountain ecosystems play a key role in sustaining local wildlife and plant diversity and ecosystem processes such as material - energy cycle, soil and water conservation.

Spatial distribution of ecological dynamic resistance (Figs 1c and S7, S8) was calculated using a function of ecological adaptation resistance based on ecological resilience, ecological sensitivity, and human disturbance intensity. When NDVI and dynamic resistance results were compared, both spatial distribution patterns were similar in that the resistance of the western and central regions was higher than that of the eastern region (Fig. 1c). Only 6.68% of the study area corresponded to the highest category of ecological dynamic resistance, while the first and second categories accounted for 48.42% (Table S3). Zhangjiakou, a rapidly expanding city, had relatively high ecological dynamic resistance with 69.32% of the city area corresponding to the fourth and fifth categories, which was a result of high human disturbance intensity and imperfect ecosystem resilience. Furthermore, the mountainous area with high vegetation coverage and less human disturbance showed lower ecological dynamic resistance, for example, in Zhuolu and Chongli County. However, a large number of ski venues will be constructed in Chongli County in the near future, which will inevitably lead to weakened ecosystem resilience and an increase in the trend of human interference.

### Ecological resistance for Olympic construction region in Chongli District

The Sitaizui County and Xiwanzi County are key construction areas for winter Olympic venues and supporting facilities. However, the ecological function resistances of Sitaizui County and Xiwanzi County were at high levels, with the fourth and fifth categories together accounting for 72.67% and 77.25%, respectively, indicating that most areas were not suitable for large-scale construction and development. Ecological structure resistance in Sitaizui and Xiwanzi in the first and second levels account for 30.29% and 28.94%, respectively, while ecological dynamic resistance remains at lower levels with the first category accounting for 73.02% in Sitaizui and 69.15% in Xiwanzi (Tables S4–S6). On the whole, structure resistance in the western area (Shizuizi and Yimatu counties) was less than in the eastern area (Fig. S9), and function resistance in the northern area was less than in the southern area (Fig. S10). In Chongli, many areas have relatively low dynamics resistance due to a high degree of vegetation dynamics and low intensity human disturbance (Fig. S11).

To further study the combined ecological resistances in Chongli County, the ecological resistances were divided into three categories, with the first and second level merging into the lower grade, the third level for the middle grade, and fourth and fifth level for the higher grade. Then, the combination of ecological resistance for regional construction was classified into five categories as shown in Fig. 1d. The results showed that high combination levels (level 4: two-high; level 5: three-high) with high ecological resistance occupy 47.91% of the Chongli area. These areas with high ecological constraints are not suitable for large-scale construction. Only 9.51% of the area in Chongli has conditions suitable for construction. The areas of Sitaizui and Xiwanzi, with a high combination of levels, account for 67.87% and 69.09%, respectively, also indicating a high ecological resistance for intensive construction.

### Ecosystem damage assessment in the Winter Olympic ski area

A high-profile Olympic tourist and ski area, located in the northern mountainous area in the Yanghe watershed, was taken as an example to study the ecosystem loss. Ski construction will lead to the occupation of land resources, the destruction of forest and grassland and the extraction of groundwater, which will inevitably produce a series of ecological impacts. It will primarily change the land use pattern, decrease the biomass, cause the migration of animals, result in the loss of soil and water, result in desertification, reduce groundwater resources, and so on. These factors directly interfere with the integrity and stability of the local ecosystem by the damaging ecological structure, ecological function and ecological dynamics.

The spatial distribution of the damaged ecosystem is directly related to the construction scale and the geographical location of the ski resort. The total loss of the natural ecological system in the ski area is 117.27 hm^2^, accounting for 28.6% of the whole natural area, which is divided into 14 target planning areas corresponding to five functional areas (Table S9). The ecological system area damaged by functional construction included the construction area, infrastructure project area, the main project ski area, service facilities construction area and the service security system area, and accounted for 16.94%, 4.76%, 4.55%, 1.78% and 0.56% respectively. Among the 14 target planning areas (Table S9), the field of play is the largest (2.67%), the parking lots being most widespread and accounting for 2.49%. The damaged forest ecosystem is 66.11 hm^2^, accounting for 28.79% of the total forest area; shrubs are 7.48 hm^2^, accounting for 15.67% of the whole shrub area; the loss of meadows is 29.9 hm^2^, occupying 31.96%, and the total loss of barren land is 13.78 hm^2^, which has a destruction ratio as high as 83.15%. Considering the absolute destruction of vegetation on the ski resort, the most severely damaged ecosystems are the forests, meadows and shrubs in descending order.

**Figure 2:**
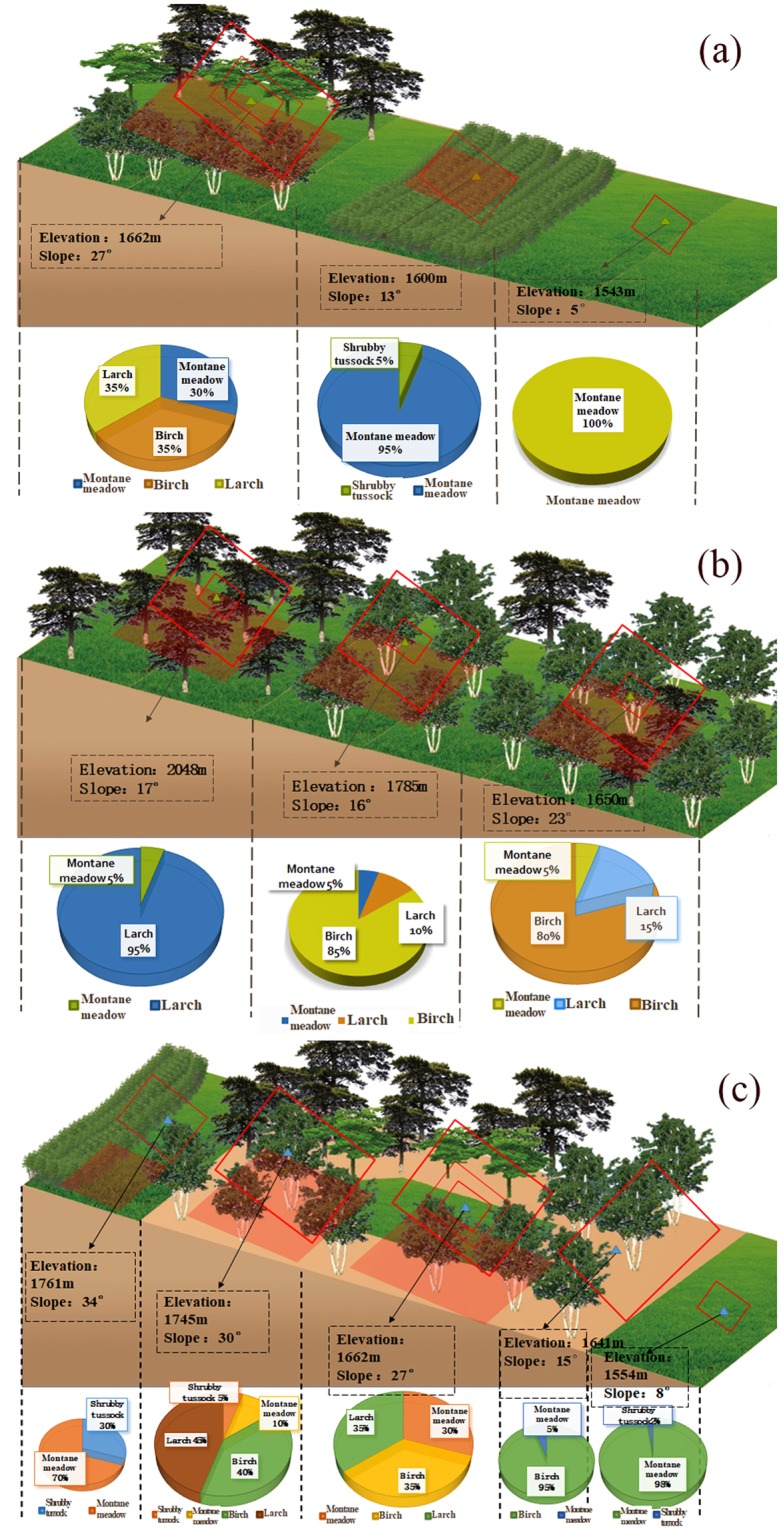
Vegetation cover of different ski levels. (**a**) Primary ski piste; (**b**) Intermediate ski piste; (**c**) Advanced ski piste.

From an ecological perspective, the Field of Play for mountainous areas, which enables freestyle skiing and snowboarding events at the 2022 Winter Olympics, also plays a vital role in water retention, soil conservation and protecting biological diversity. To study the ecosystem function loss in different ski runs, we investigated vegetation coverage (Fig. 2) and soil conservation information in the field. The function of water retention, soil conservation, and biomass accumulation are taken into account by the four types of vegetation (Table 1). Generally, the logging area in various ski categories numbered 10.7 hm^2^ and the permanent biological loss could be up to 3,304.9 t/hm^2^·a (fresh weight) in total. The large amount of deforestation required to build multiple ski runs increases soil erosion, destroys plant life, reduces plant species diversity, and causes permanent damage to the ecological environment of the surface soil. Despite having more abundant herbaceous species on primary ski pistes than on others (Fig. 2), the indicator values of the vegetation composition showed an increased supply in moisture and nutrient retention (N, P_2_O_5_, and K) available for plants from primary to advanced ski pistes. In terms of coverage and growth status, more biomass accumulation and soil reinforcement were found in advanced ski pistes. Additionally, the variations of soil physical and chemical indicators (including bulk density, moisture content, visible erosion, and nutrients) may directly relate to the changes of multiple ecosystem functions. The results also indicated that the soil moisture content increased and that the soil bulk density decreased with increasing distance from the construction areas.

**Table 1:** Ecosystem function loss of different levels of Olympic ski pistes.

Ski level	Vegetation	Loss area (Hectare)	Water retention	Soil conservation				Biomass accumulation	
			Regulating water (m^3^/hm^2^·a)	soil reinforcement (t/hm^2^·a)	N (kg/hm^2^·a)	P_2_O_5_ (kg/hm^2^·a)	K (kg/hm^2^·a)	fresh weight (t/hm^2^·a)	dry weight (t/hm^2^·a)
Primary ski piste	Birch	0.2	130.2	7993.7	9.9	1.8	1.8	109.6	—
	Larch	0.2	94.5	6	7.9	3.8	4.5	149.7	—
	Shrubby tussock	0.1	56.1	5.2	7.1	2.3	—	—	—
	Montane meadow	1.9	892.6	162	279.3	56.6	—	15.5	4.2
Intermediate ski piste	Birch	2	1431.2	87885.1	108.3	19.6	19.8	774.1	—
	Larch	0.6	253.8	15.9	21.2	10.3	12.1	921.8	—
	Shrubby tussock	1.1	757.1	70.2	95.5	31.7	—	—	
	Montane meadow	1	452.8	82.2	141.7	28.7	—	25	7.4
Advanced ski piste	Birch	3.1	1910.5	117331.6	144.6	26.2	26.4	379.7	—
	Larch	4.6	1815.9	114.4	151.4	73.8	8.6	884.8	—
	Shrubby tussock	1.7	1239.2	114.7	156.9	50.8	—	—	—
	Montane meadow	0.4	166.5	30.2	52.1	10.6	—	44.7	10.5

## Discussion

**Figure 3:**
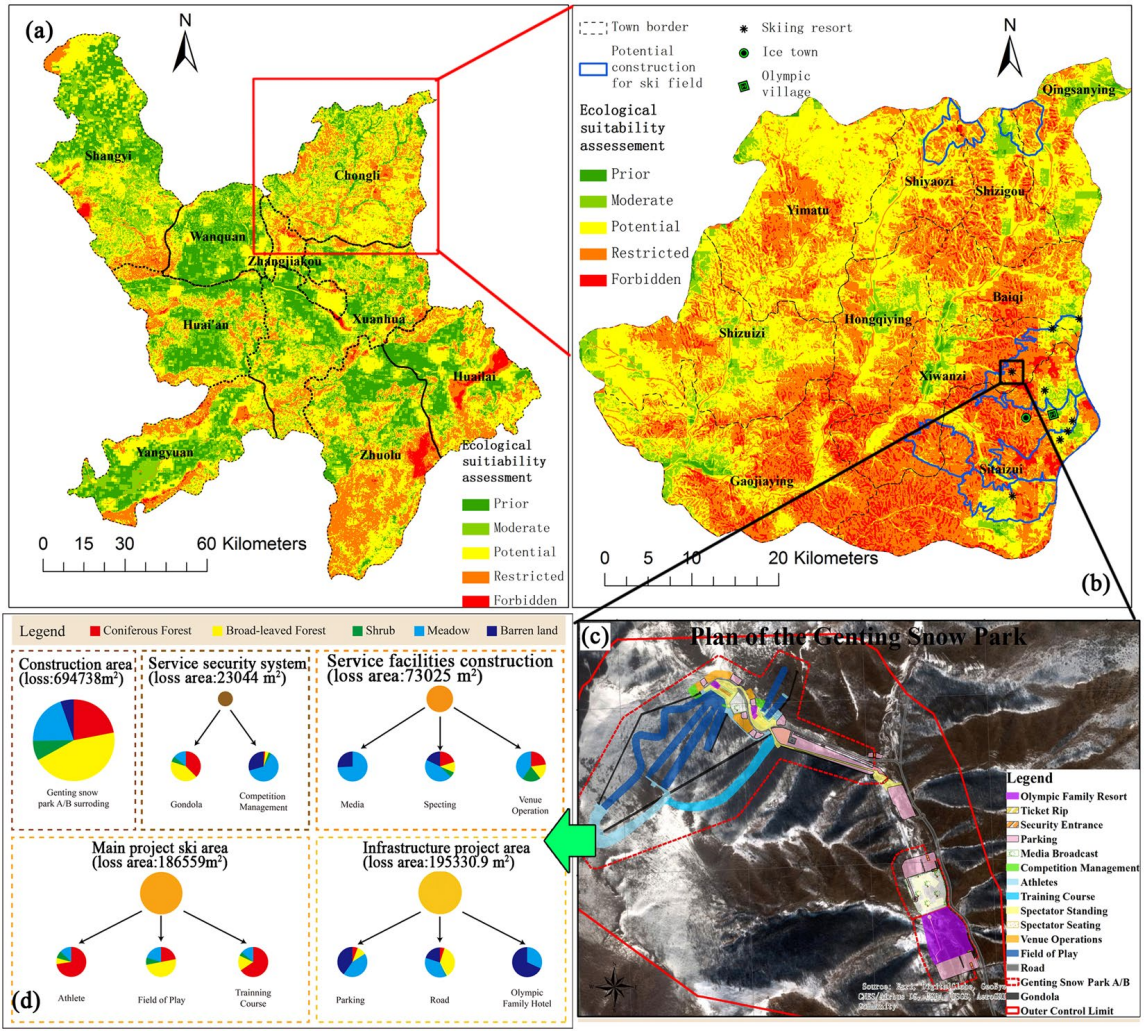
Ecological suitability assessment for construction in Yanghe Basin (**a**) and Chongli District (**b**), functional area distribution of the constructing Olympic ski pistes (**c**) and different types of ecosystems loss area (**d**). The figure (**a**–**c**) is generated by ArcGIS 10.1 software, http://www.esri.com.

There is a huge conflict between the regional construction and ecological loss due to the current and future development required by the Winter Olympics^13,40^. Land and economic development are accompanied by landscape changes and ecological destruction in the cities that hold the Olympics^6,16^, and the 2022 Winter Olympic Games could also bring large-scale regional development and population aggregation in the study area. However, at a regional scale, the spatial ecological suitability results show that the areas with lower integrated ecological resistance are mainly located in plains along the Yanghe River, including Shangyi, Wanquan, Zhangjiakou and Xuanhua counties, where construction will be less of a disturbance to the eco-environment (Table S8). The prior and moderate disturbance zones (Fig. 3a) for Chongli are smaller, compared to the other counties. In Chongli County, the areas suitable for large-scale development and utilization are limited in the towns of Xiwanzi and Sitaizui (Table S7), where the Winter Olympic venues and supporting facilities are located (Fig. 3b). Therefore, the ecological suitability of concentrated deployment for major construction in Chongli should be considered, and the forbidden development zone mainly distributed in natural conservation areas and some other well-covered mountainous regions, where all development activities should be prohibited. Basic farmland^28^ should be strictly protected, and key ecosystems should be maintained effectively in restricted and potential zones in Chongli.

For the Winter Olympic venues, the ecological indicators that have relatively low ecological resistance include the slopes, DEM, general geological hazards, earthquake, and vegetation stability. However, the indicators, such as biodiversity protection, water retention, and soil conservation, have high ecological resistance to the ski resort we studied. Hence, a sensitivity analysis of all the parameters controlling suitability results was conducted, with one sub-region (N40.967°, E115.386°) chosen as an illustrative example. The results of sensitivity analysis indicated that the weightings of ecological structures (*w*_*s*_), functions (*w*_*f*_), dynamics(*w*_*d*_), water retention, soil conservation, earthquake, and DEM were the most sensitive parameters (Table S11), which might be the main source of uncertainty of modeled suitability grades in the studied region. Furthermore, compelling evidence was found that the impact on the ecosystem increases with the increased severity of disturbances associated with Winter Olympic construction. For example, the positive effects of the migration of industrial structures and the negative effects of permanently disappearing woodland and grassland due to constructions will influence the stability of the local ecosystem. Additionally, infrastructure construction fragments the regional coniferous forests, broad-leaved forests, shrubs, meadows and barren ecosystems. Due to unreasonable land utilization, forests, shrubs and grasslands have been seriously damaged in concentrated construction (Fig. 3c,d), which weakens soil retention capacity and intensifies soil erosion. Meanwhile, changes in the vegetation structure disturb animal habitats, which may lead to migration or population loss. This migration may give rise to the potential for interspecies competition. The deteriorated forest ecosystem may lose the function of precipitation interception and storage, increase the risk of landslides, collapse, debris flow and other geological disasters, and result in catastrophic effects on regional ecological security. For the site-specific construction, we suggest that (1) the projects that have been built or are being legally constructed should further strengthen efforts made towards ecological restoration, for example, the restoration of herbaceous plants on the ski pistes in the summer, the construction of ecological corridors, and the strengthening of water conservation measures. (2) Local land use planning should be changed for better coordination and consistency of policy-making in regional development and ecological preservation. (3) The government should consider new projects to be constructed in the areas with less ecological resistance. To further reduce the loss of natural complex ecosystems, each skiing area must be reasonably planned, on a limited scale, to resolutely put an end to disorderly development. (4) Ecological *in-situ* monitoring networks should be strengthened. (5) The accommodation, amusement and other supporting facilities of the Winter Olympics are recommended to move to Chongli and Zhangjiakou urban areas or regions with prior development.

At the regional scale, ecosystem services and economic development are a mutual-constraining paradox^4^ that is particularly prominent in the Yanghe Basin. The core benefits of the cooperation between Beijing and Zhangjiakou would not be the same. Poverty alleviation and development are still the first requirement in Zhangjiakou; however, in order to improve the water and air environment^41,42^ for the lower Yanghe basin and Beijing, more than 600 higher polluted enterprises were closed in Zhangjiakou. Undoubtedly, the Winter Olympics could bring a series of local benefits, including economic and social benefits, eco-city construction and effective branding. Similarly, it will stimulate market consumption demands and tourism, especially in the ski industry. However, for the whole basin, due to the vigorous expansion of the tourism industry driven by the Winter Olympics in Chongli, it will inevitably lead to continuous emissions in the region upstream and increase the pressure to protect the source of water downstream in the future. Additionally, the vegetation destruction and soil erosion occurring over the soil surface, which results from rainwater and snowmelt in spring and summer, are going to generate a high content of N, P loss on the steep slopes. Accordingly, soil and water conservation measures at different scales are vital to control non-point source contamination, protect water source quality and ensure drinking water security. Furthermore, multi-scale balancing of conservation and development, especially for the key indicators^43–45^, should be carefully considered by the government. The development priority levels were considered to be suitable for concentrated constructions, and the forbidden zones need to be protected and all forms of land use conversion should be prohibited. Ecological legislative protection should be strengthened, and ecological compensation should be implemented to reduce the conflicts between space planning and land use. Therefore, the feasibility and implementation strategy of building an integrated ecological function area for the Yanghe basin is very important to promote the steady economic growth and improve the ecological environment.

## Materials and Methods

### Study area

The Yanghe basin, located northwest of Beijing, is the host of the 2022 Winter Olympics, and it occupies a vital strategic position as an important water resource and an ecological protection barrier for Beijing. Zhangjiakou is the central area of economic development in the Yanghe basin. Three tributaries converge and flow into Yanghe River at Huai’an County, with a watershed area of approximately 14,600 km^2^. The Yanghe River flows through Wanquan County, Huai’an County, Zhangjiakou City, Xuanhua District, Xiahuayuan District, and Huailai County and flows into the Guanting reservoir, of which the mainstream length is 106 km, and the shape extending from east to west is longer than its north-south orientation. Hills and mountains account for 80% of the whole Yanghe basin, and soil and water loss is scattered in the vast regions of mountainous areas. The terrain is characterized by high hills in the west and low valleys in the east, with the elevation in the northeast and northwest reaching the altitudes of 1,200–1,500 m, while the western and southern areas range between 500–1,000 m in elevation. The main soil type is the mountain yellow sandy loam soil, followed by sandy gravels and yellow clay soils in some local areas.

Chongli District, with a total area of 2,334 km^2^ and a population of 0.126 million, is located in the northwest of Hebei Province as the transition zone between the Inner Mongolia plateau and the North China Plain. This county contains 10 towns and more than 400 villages. The elevation ranges from 814 m to 2,174 m. Mountainous areas in Chongli are widely distributed, accounting for 80% of the whole territory, with a high forest cover rate of 52.38%. The average temperature is −12 °C in the winter, and there is an abundant negative oxygen ion concentration, more than 10,000/m³ was found in the air. Genting Snow Park is located in the northern area of Chongli District, which is among the Dama mountains, on the border of Taihang and the Yanshan mountains. It is a typical mountainous area located in the East Asian continental monsoon climate in the Asian temperate arid region, with an average annual temperature of 3.3 °C and a typical snowfall period of 150 days. According to climate and terrain conditions, the regional ecological system contains streaky meadow ecosystems, deciduous broad-leaved forest ecosystems, coniferous forest ecosystems, alpine shrub ecosystems and alpine meadow ecosystems, ranging in altitude between 1,700 and 2,100 m. The ski resort is dominated by cinnamon soil and brown soil in the mountain forests, where 62 species of trees account for 11.2% of total species in the studied region, belonging to 75 families and 132 genera. Specifically speaking, trees with birch and larch, bushes with Spiraea and Rosaceae, and herbaceous species have a higher coverage on piste plots than on other areas.

### Ecological field survey

We conducted field measurements between 6 June and 12 October 2016 in a ski resort, during peak blooming periods and before the onset of leaf senescence. Moreover, we used a blocked sampling design within each ski run area. According to the altitude and the type of ecosystem, we focused on the sample survey in the primary, intermediate and advanced ski pistes. A total of 21 sample plots were set up for this study. Among them, the primary ski piste contained one arbor quadrat, one shrub quadrat and three herb quadrats. The intermediate ski piste was arranged with three arbor quadrats, three shrub quadrats and four herb quadrats, while the advanced ski piste contained three arbor quadrats and three herb quadrats. The sizes of arbor, shrub and herb quadrats were set to 20 m*20 m, 5 m*5 m, and 1 m*1 m, respectively. The vegetation types, vegetation coverage, biomass, ecosystem diversity index, and slopes were investigated. The area of ecological function loss was estimated by combining survey data and remote sensing data. In addition, the concentrations of the total nitrogen in soils were analyzed in the laboratory using the Kjeldahl digestion and steam distillation method. The exchangeable potassium was extracted by ammonium acetate and determined by flame photometer. The phosphorus was analyzed by the alkali fusion-Mo-Sb Anti spectrophotometric method (HJ632-2011, Ministry of Environment, China).

### Data sources and ecological suitability evaluation

An ecological suitability evaluation for Winter Olympic construction in mountainous areas involves datasets on topography (DEM), land use, geological disasters (landslides, debris flows, geological fracturing, and subsidence disasters), vegetation (NDVI), soil, precipitation, population density, GDP per area, and other factors (Fig. S12). Most of the above data was collected from 2013 to 2015 to minimize the variability due to long-term data changes, and data source details are shown in Table S10.

Winter Olympic construction is a process of human activity to overcome the resistance of natural ecosystems for sustainable development. Then, the integrated ecological resistance (IER) has a negative correlation with the level of ecological suitability, which means that the higher the former, the lower the latter. There is a high correlation between construction security risks and ecological risks, which have been calculated and graded into several categories separately to characterize the resistance of ecological structures (*ES*), ecological functions (*EF*), and ecological dynamics (*ED*). Peng’s conceptual model was described as follows^10^:

1 $$IRE=ES\times {w}_{s}+EF\times {w}_{f}+ED\times {w}_{d}$$

where *w*_*s*_, *w*_*f*_, and *w*_*d*_ are the weights of the indies. According to the literature^10^, given that structures, functions, and dynamics are interdependent for the entire ecological environment, these three weights were given equal values. Based on regional characteristics, we defined representative indicators and calculation methods. Integrated ecological resistance was evaluated as indicated in Table S10. Details of individual resistance methods were shown in SI Methods. In summary, the ecological structural resistance was determined by elevation, slope, and other terrain conditions, as well as the geological disaster frequency, earthquake frequency, and distance to fracture zones^46^. The weight of each structural index was calculated by the method of Analytical Hierarchy Process (AHP). The ecological function resistance was evaluated by biodiversity protection, water and soil conservation. The classification and weightings of biodiversity protection and water retention refer to the methods in the literature^10,45,47^. Soil conservation was evaluated by the revised universal soil loss equation (RUSLE). Ecological dynamics assessment was reflected by vegetation stability (expressed by normalized difference vegetation index), ecological sensitivity^48^ (expressed by the distance to sensitive areas), and human disturbance (expressed by spatial population density). Generally, the more stable the geology and the flatter the terrain, the lower the ecological resistance to Winter Olympic development by assessing ecological structure resistance. The higher the level of ecological importance, the greater the ecological risk of mountainous area development by determining ecological functional resistance. If the negative relationship between construction sites and ecological function indicators was found, which would lead to the decline of ecosystem service functions. The construction of venues has increased the intensity of disturbances in regional human activities, resulting in a decrease in vegetation stability, leading to ecological fragility and sensitivity. Meanwhile, higher ecological resilience from the ecological dynamic assessment indicates a greater capacity for self-recovery and self-updating to counteract disturbance or damage, as well as less ecological risk and lower ecological resistance to construction.

The sensitivity analysis of the main indexes and weightings was conducted. For each evaluation, one parameter was selected and increased by 1%. The sensitivity coefficient S of this parameter was defined using formula (2):

2 $$S=(Y1.01-Y)/(0.01\times Y)$$

where Y was the original model output, and Y1.01 was the model output with increased parameter

## Electronic supplementary material

Supporting materials
