# Supplementary material for: Balancing conservation and development in Winter Olympic construction: evidence from a multi-scale ecological suitability assessment
Source: Sci Rep. 2018 Sep 20;8:14083. doi: 10.1038/s41598-018-32548-2 (PMC6147948; doi:10.1038/s41598-018-32548-2)
Supplement: Supplementary file 1 — Supporting materials [file 41598_2018_32548_MOESM1_ESM.docx]

**Balancing conservation and development in Winter Olympic construction: evidence from a multi-scale ecological suitability assessment**

Shuai Song^a,c,+^, Sheng Zhang^b,+^ , Tieyu Wang^a,c,*^ , Jing Meng^a^,

Yunqiao Zhou^a,c^, Hong Zhang^d^

^a^ State Key Laboratory of Urban and Regional Ecology, Research Center for Eco-Environmental Sciences, Chinese Academy of Sciences, Beijing 100085, China

^b^ School of Environmental & Natural Resources, Renmin University of China, Beijing, 100872, China

^c^ University of Chinese Academy of Sciences, Beijing 100049, China

^d^ College of Environmental & Resource Science, Shanxi University, Taiyuan 030006, China

+ These authors contributed equally to this work and should be considered co-first authors

* Corresponding author:

Tieyu Wang, State Key Laboratory of Urban and Regional Ecology, Research Center for Eco-Environmental Sciences, Chinese Academy of Sciences, No.18, Shuangqing Road, Haidian, Beijing 100085, E-mail address: wangty@rcees.ac.cn

**Contents**

[Figure S1 Spatial resistance patterns of ecological structure factor Slope in Yanghe basin (Generated by ArcGIS 10.1 software, http://www.esri.com). 4](#_Toc520935015)

[Figure S2 Spatial distribution of geological hazards, fracture zones, protection zones and historical earthquake locations as ecological structure factors for calculating ecological resistances (Generated by ArcGIS 10.1 software, http://www.esri.com, data source: Zhangjiakou Municipal Bureau of Land and Resources). 5](#_Toc520935016)

[Figure S3 Spatial resistance patterns of ecological structure factor DEM in Yanghe basin (Generated by ArcGIS 10.1 software, http://www.esri.com). 6](#_Toc520935017)

[Figure S4 Ecological importance resistance patterns for explaining importance of ecosystem biodiversity protection in Yanghe basin (Generated by ArcGIS 10.1 software, http://www.esri.com). 7](#_Toc520935018)

[Figure S5 Spatial resistance patterns of soil conservation in Yanghe basin (Generated by ArcGIS 10.1 software, http://www.esri.com) 8](#_Toc520935019)

[Figure S6 Spatial resistance patterns of water conservation in Yanghe basin (Generated by ArcGIS 10.1 software, http://www.esri.com). 9](#_Toc520935020)

[Figure S7 Spatial resistance patterns of vegetation stability represented by NDVI in Yanghe basin (Generated by ArcGIS 10.1 software, http://www.esri.com). 10](#_Toc520935021)

[Figure S8 Population density and ecological sensitivity pionts (nature reserves and tourist spots) in Yanghe basin (Generated by ArcGIS 10.1 software, http://www.esri.com). 11](#_Toc520935022)

[Figure S9 Spatial resistance patterns of ecological structure and the potential construction for ski field generated by slope, altitude and fragmentation of landscape patches in Chongli (Generated by ArcGIS 10.1 software, http://www.esri.com). 12](#_Toc520935023)

[Figure S10 Spatial resistance patterns of ecological function in Chongli (Generated by ArcGIS 10.1 software, http://www.esri.com). 13](#_Toc520935024)

[Figure S11 Spatial resistance patterns of ecological dynamics in Chongli (Generated by ArcGIS 10.1 software, http://www.esri.com). 14](#_Toc520935025)

[Figure S12 The process of integrated ecological resistances for construction in Yanghe Basin and Chongli district 15](#_Toc520935026)

[Table S1 Spatial statistical analysis of ecological structure resistance in Yanghe Basin 16](#_Toc520935032)

[Table S2 Spatial statistical analysis of ecological function resistance in Yanghe Basin 17](#_Toc520935033)

[Table S3 Spatial statistical analysis of ecological dynamics resistance in Yanghe Basin 18](#_Toc520935034)

[Table S4 Spatial statistical analysis of ecological structure resistance in Chongli 19](#_Toc520935035)

[Table S5 Spatial statistical analysis of ecological function resistance in Chongli 20](#_Toc520935036)

[Table S6 Spatial statistical analysis of ecological dynamics resistance in Chongli 21](#_Toc520935037)

[Table S7 Ecological suitability assessment in Chongli 22](#_Toc520935038)

[Table S8 Ecological suitability assessment in Yanghe Basin 23](#_Toc520935039)

[Table S9 Different types of ecosystems loss in the Genting Snow Part 24](#_Toc520935040)

[Table S10 The weight coefficient of judgment matrix of ecological suitability evaluation in Yanghe Basin 25](#_Toc520935041)

[Table S11 The sensitivity analysis on the indexes and the weightings for one grid (N40.967°, E115.386°) 27](#_Toc520935042)

**Methods**

Assessment of ecological structure 28

Assessment of ecological function 28

Assessment of ecological dynamics 29


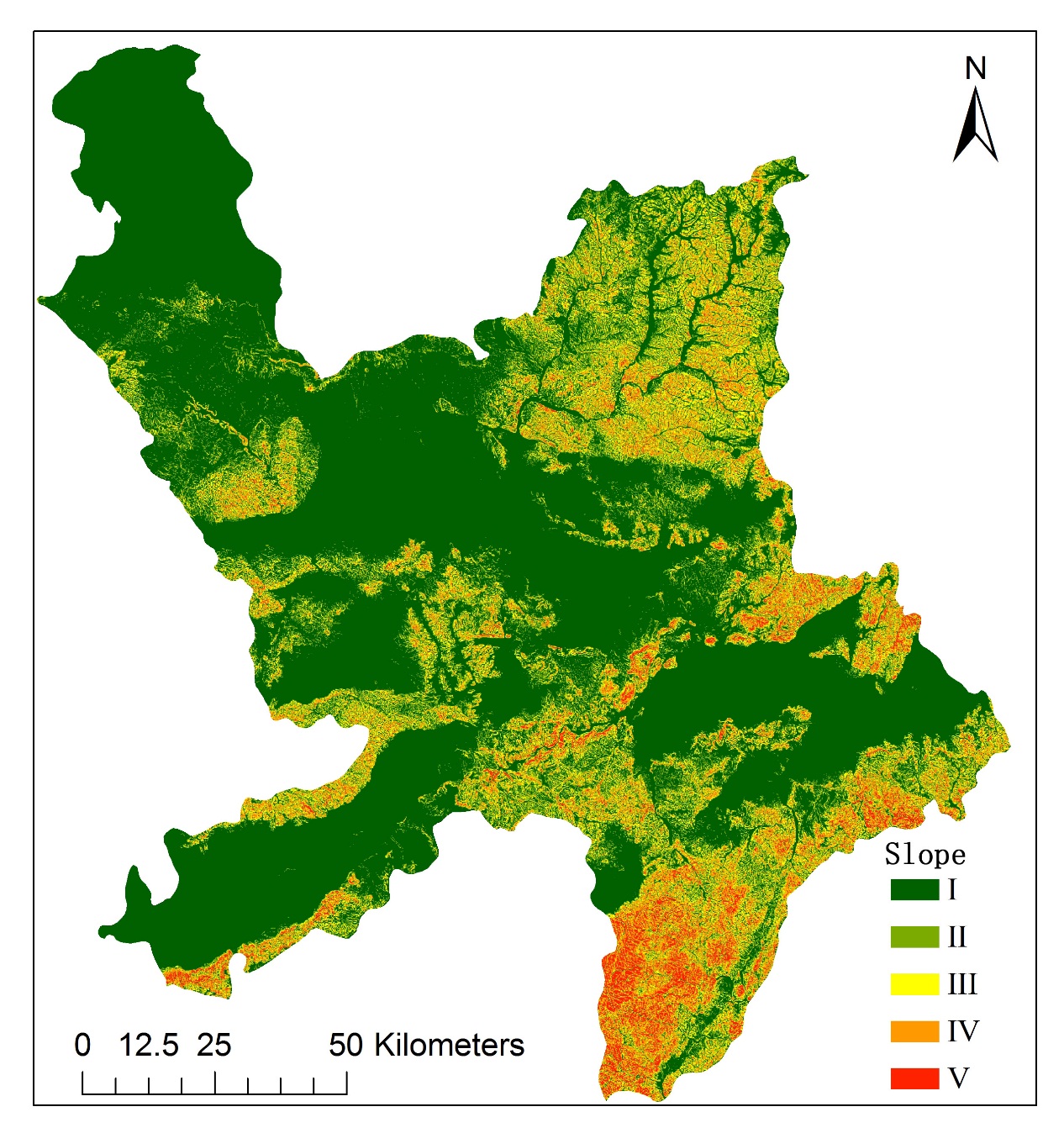


Figure S1 Spatial resistance patterns of ecological structure factor Slope in Yanghe basin (Generated by ArcGIS 10.1 software, http://www.esri.com).


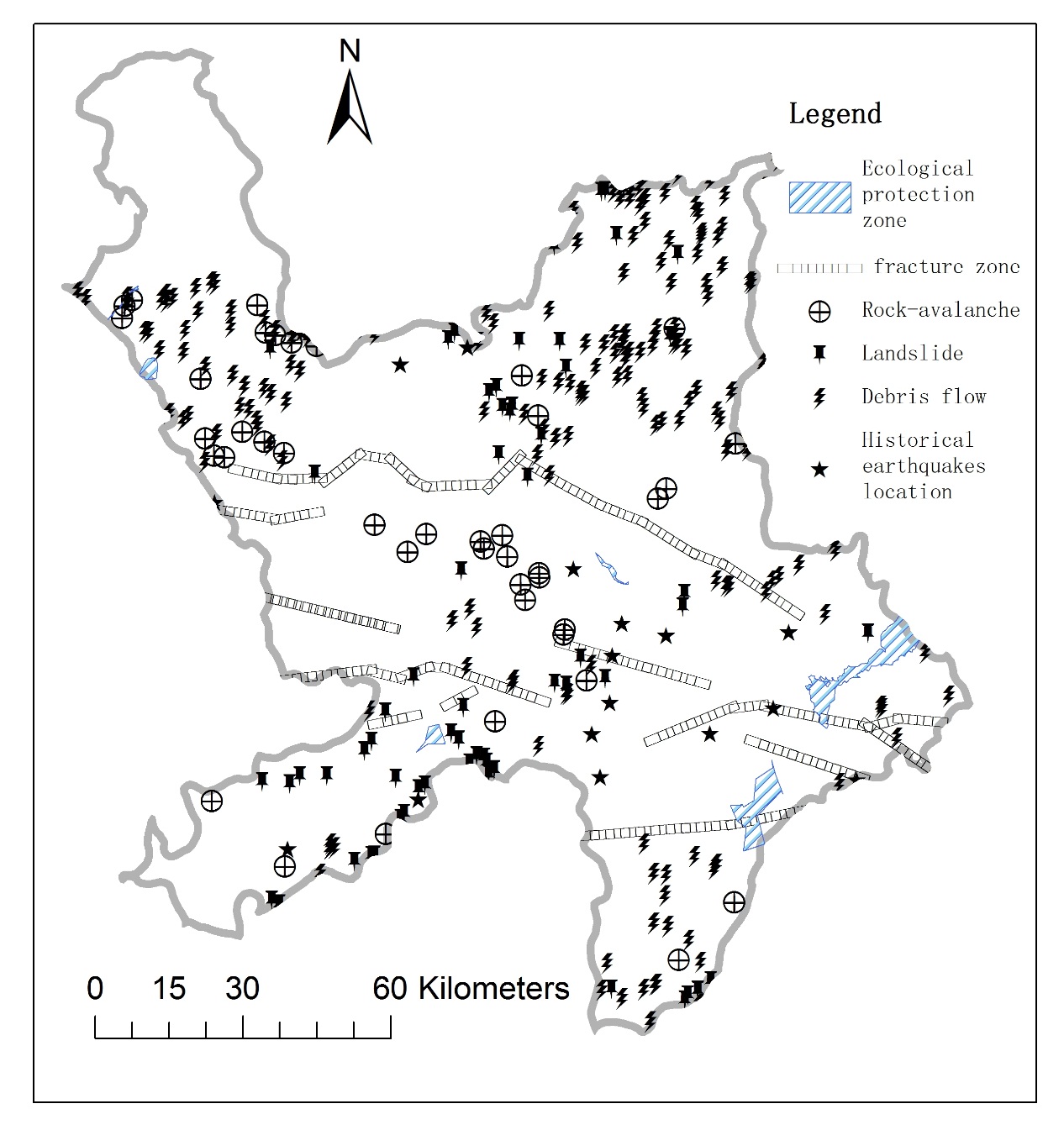


Figure S2 Spatial distribution of geological hazards, fracture zones, protection zones and historical earthquake locations as ecological structure factors for calculating ecological resistances (Generated by ArcGIS 10.1 software, <http://www.esri.com>, data source: Zhangjiakou Municipal Bureau of Land and Resources).


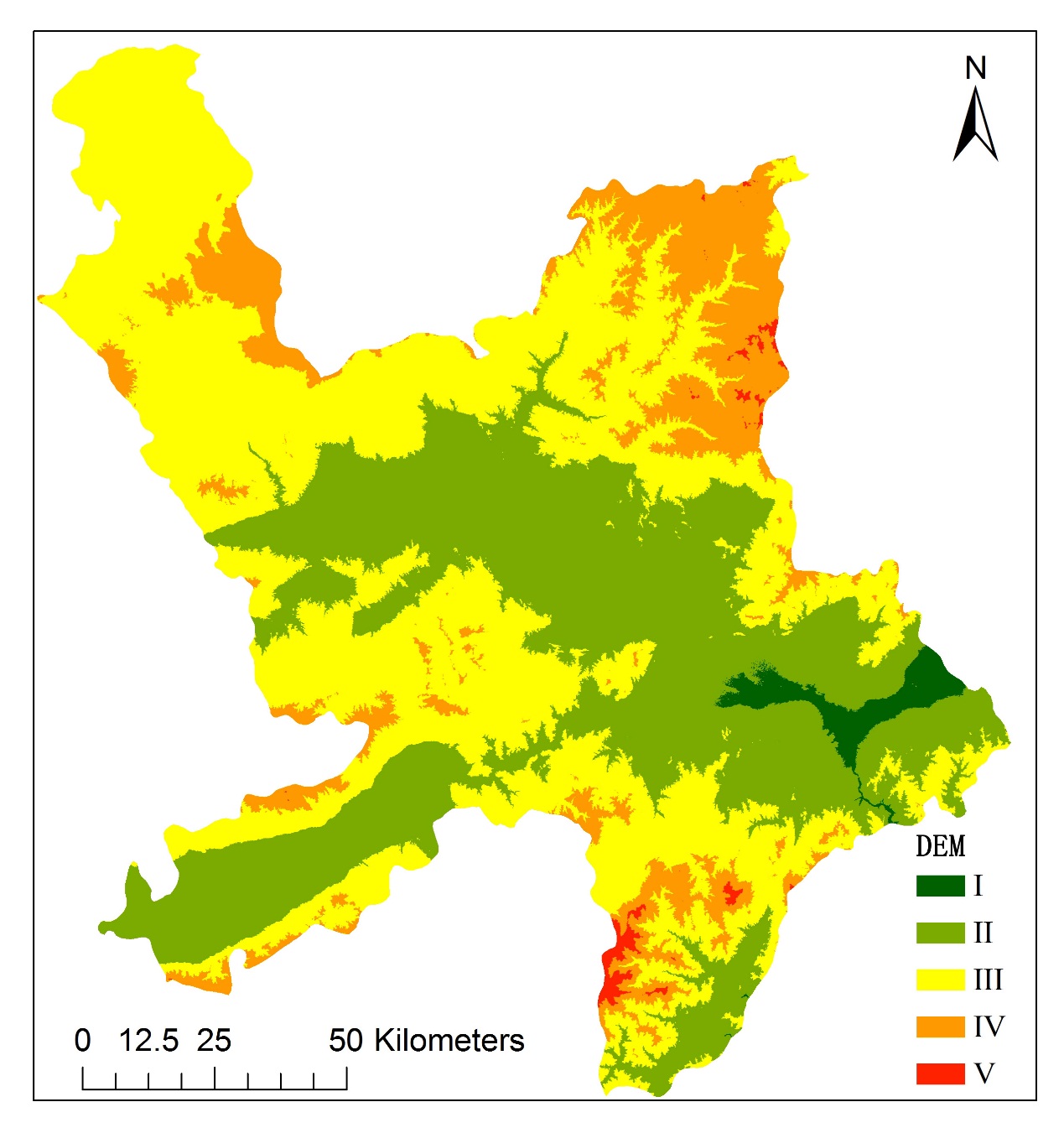


Figure S3 Spatial resistance patterns of ecological structure factor DEM in Yanghe basin (Generated by ArcGIS 10.1 software, http://www.esri.com).


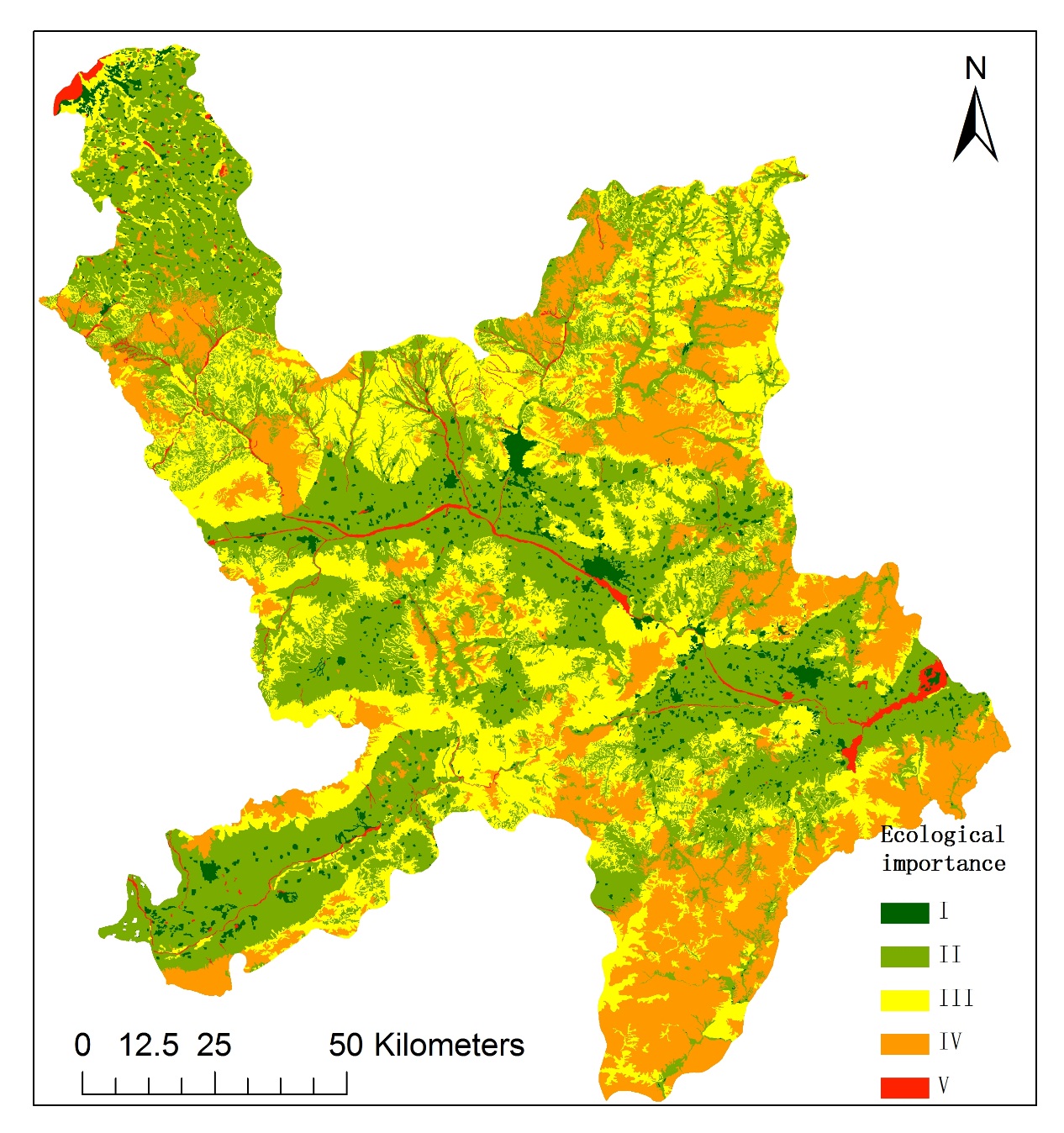


Figure S4 Ecological importance resistance patterns for explaining importance of ecosystem biodiversity protection in Yanghe basin (Generated by ArcGIS 10.1 software, http://www.esri.com).


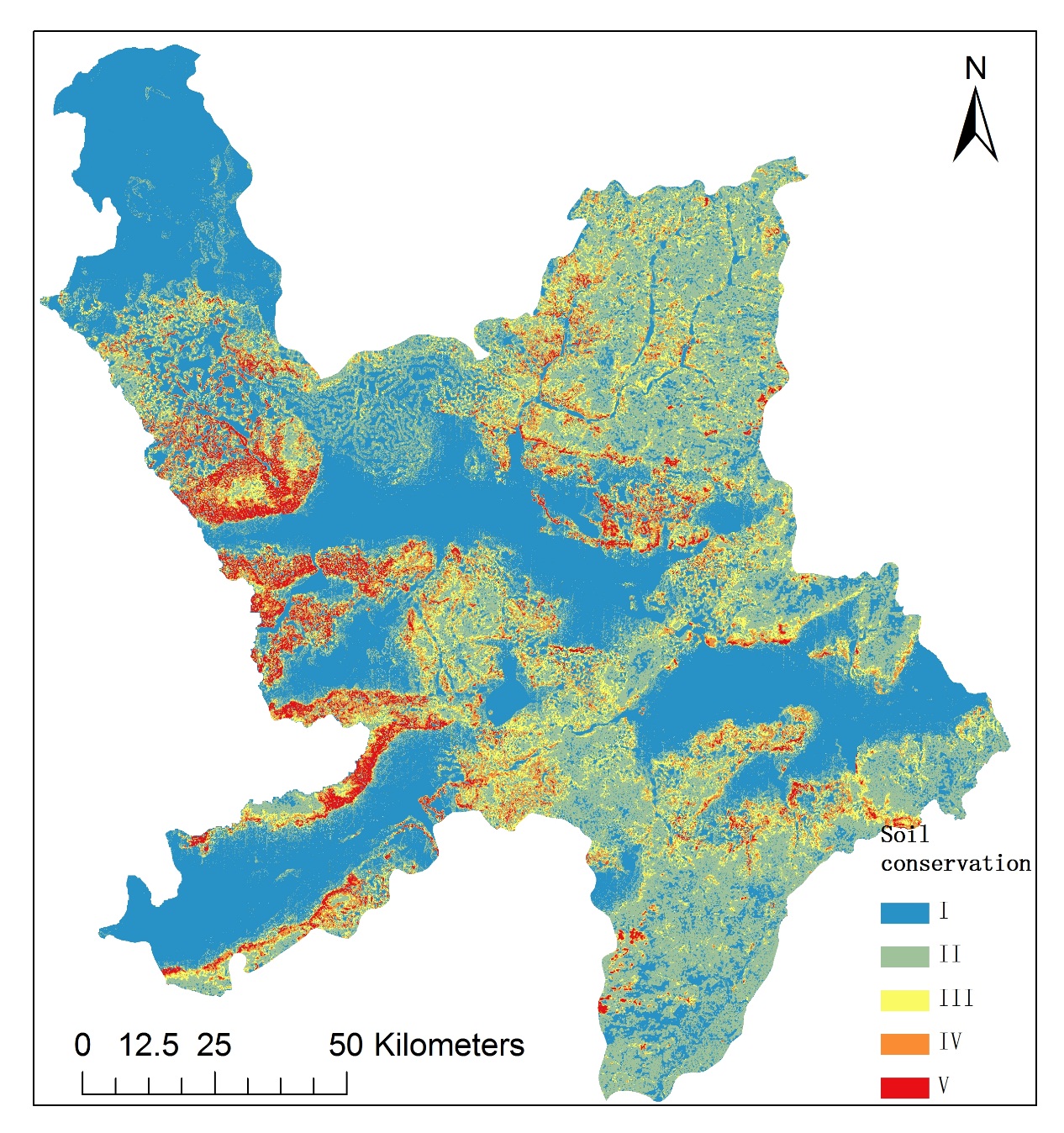


Figure S5 Spatial resistance patterns of soil conservation in Yanghe basin (Generated by ArcGIS 10.1 software, http://www.esri.com)


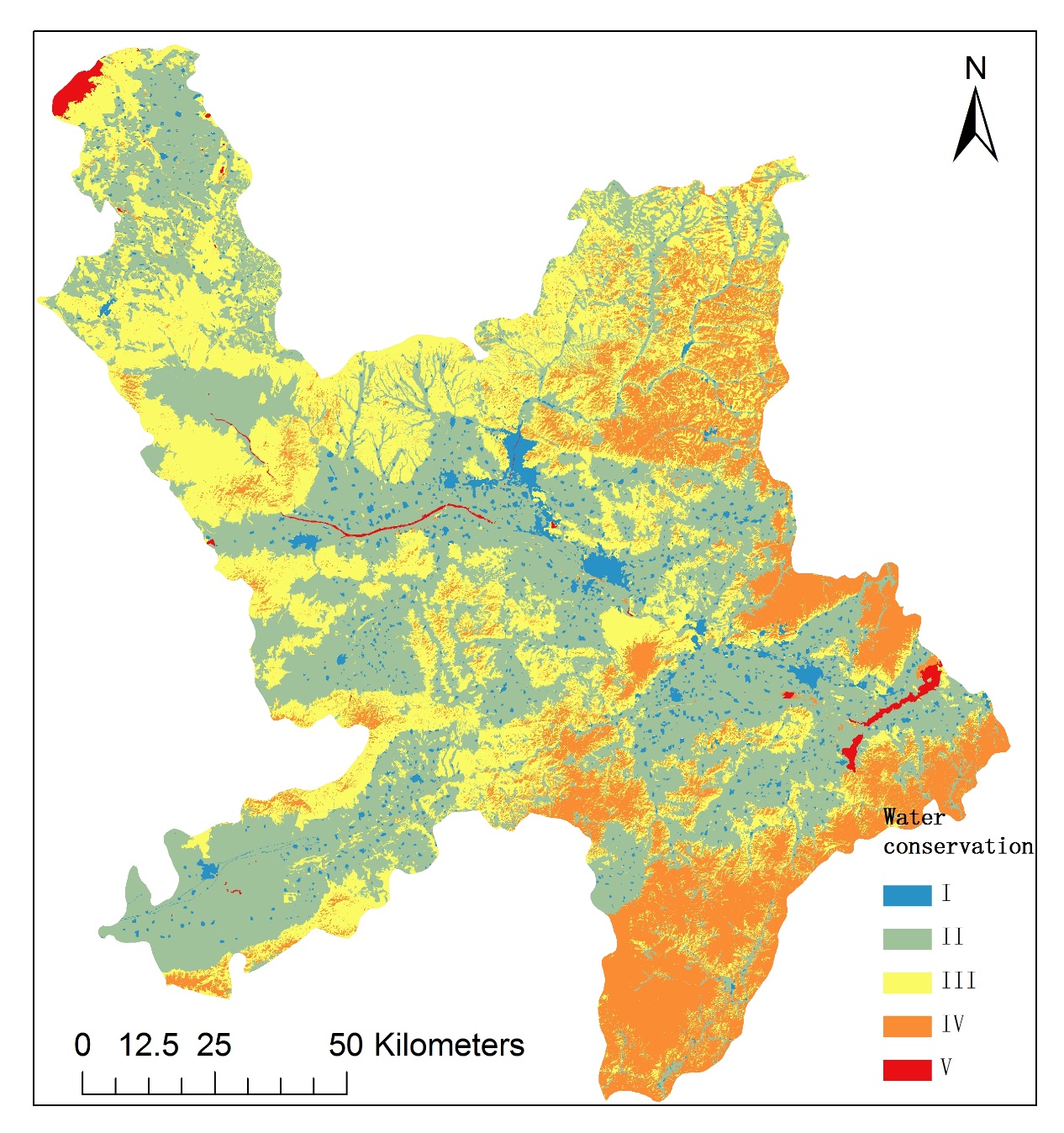


Figure S6 Spatial resistance patterns of water conservation in Yanghe basin (Generated by ArcGIS 10.1 software, http://www.esri.com).


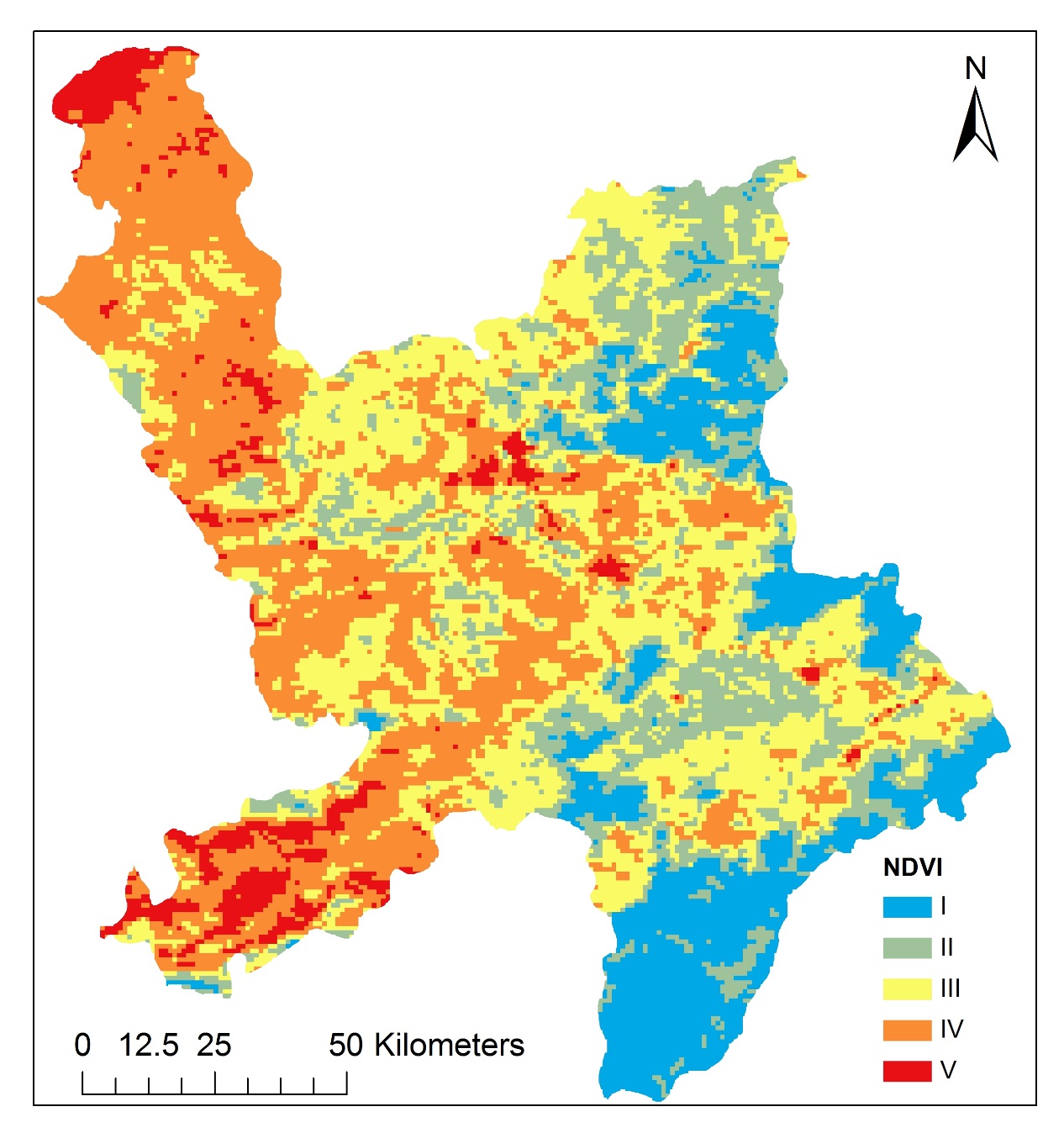


Figure S7 Spatial resistance patterns of vegetation stability represented by NDVI in Yanghe basin (Generated by ArcGIS 10.1 software, http://www.esri.com).


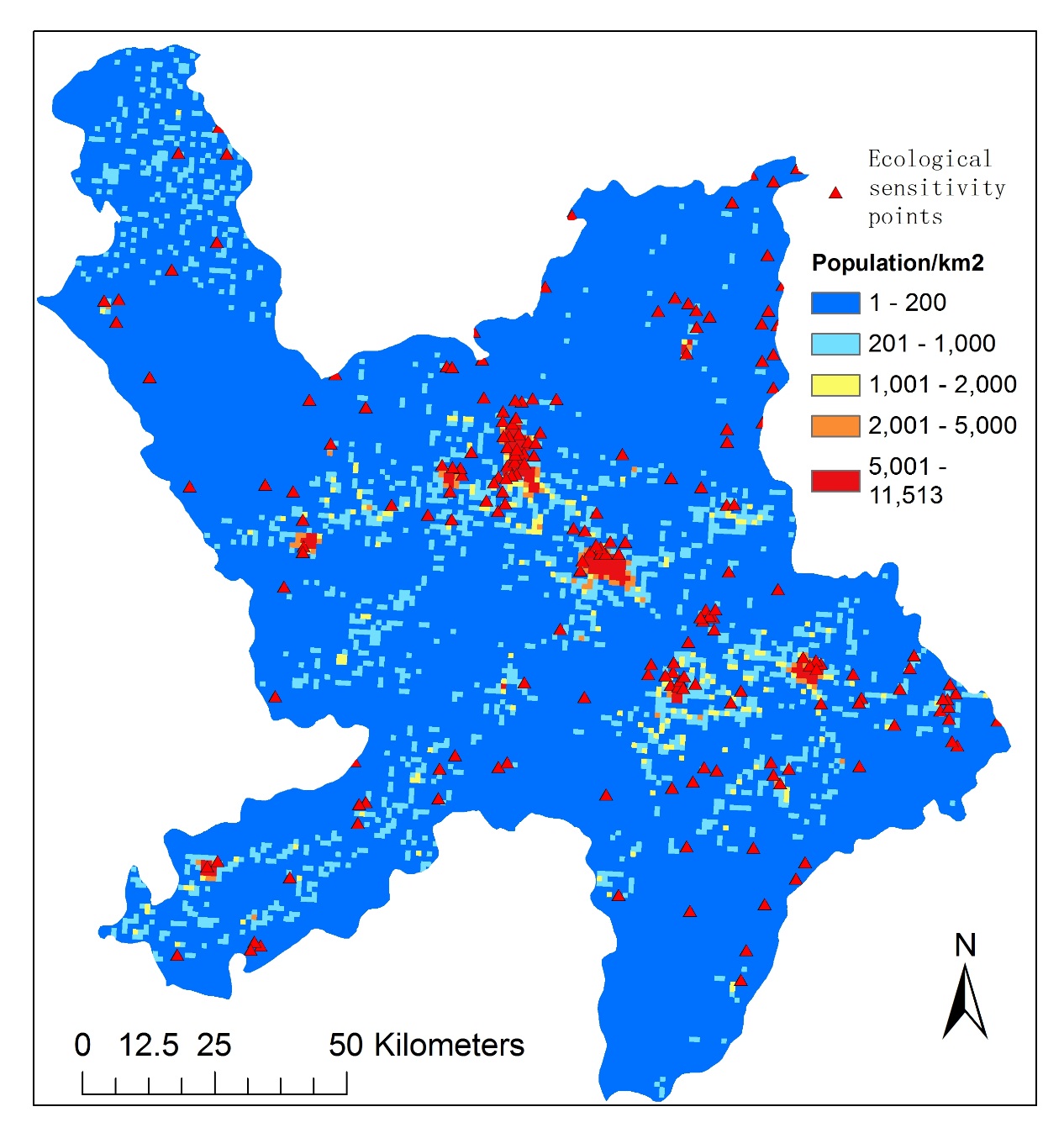


Figure S8 Population density and ecological sensitivity pionts (nature reserves and tourist spots) in Yanghe basin (Generated by ArcGIS 10.1 software, http://www.esri.com).


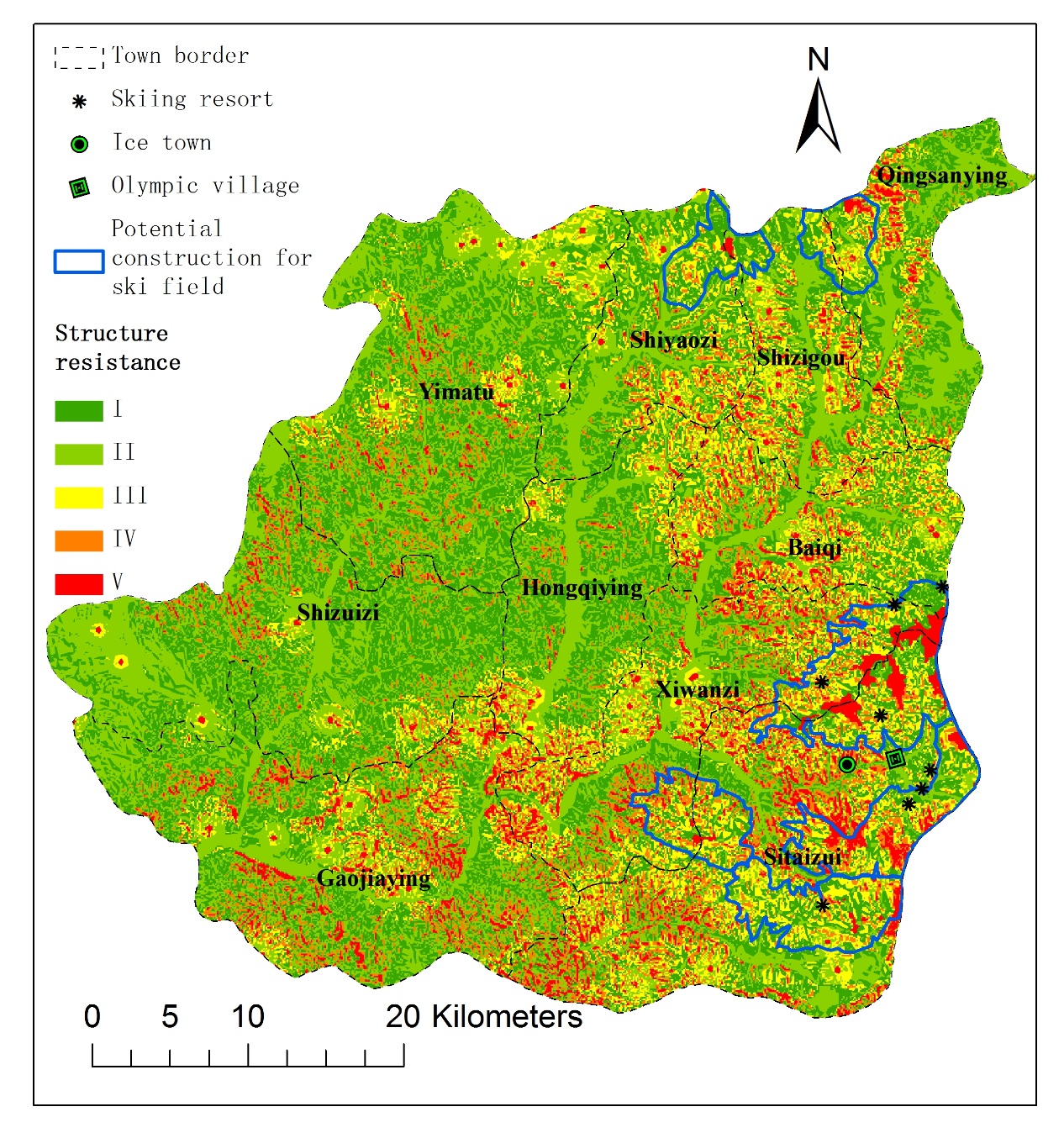


Figure S9 Spatial resistance patterns of ecological structure and the potential construction for ski field generated by slope, altitude and fragmentation of landscape patches in Chongli (Generated by ArcGIS 10.1 software, http://www.esri.com).


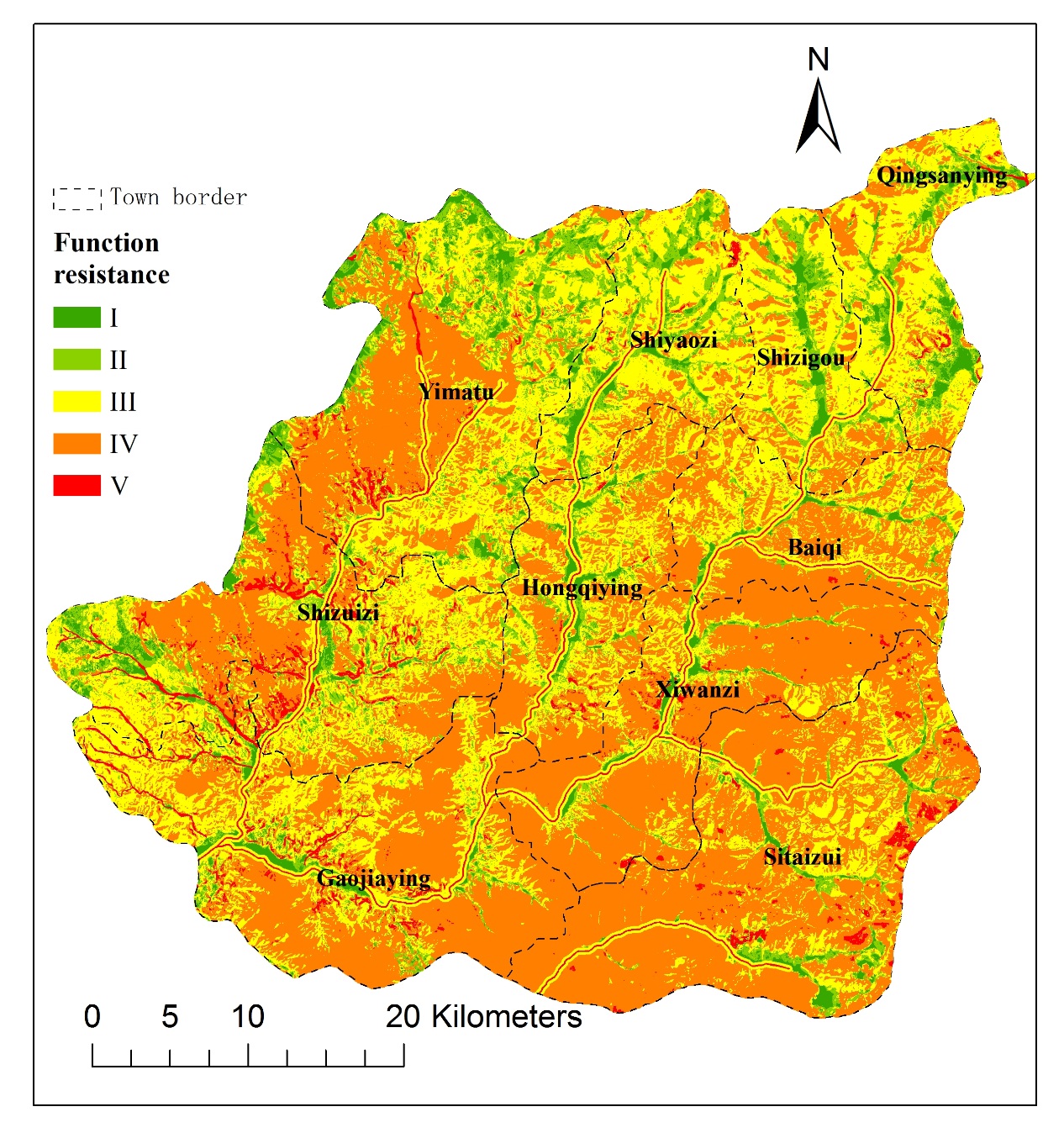


Figure S10 Spatial resistance patterns of ecological function in Chongli (Generated by ArcGIS 10.1 software, http://www.esri.com).


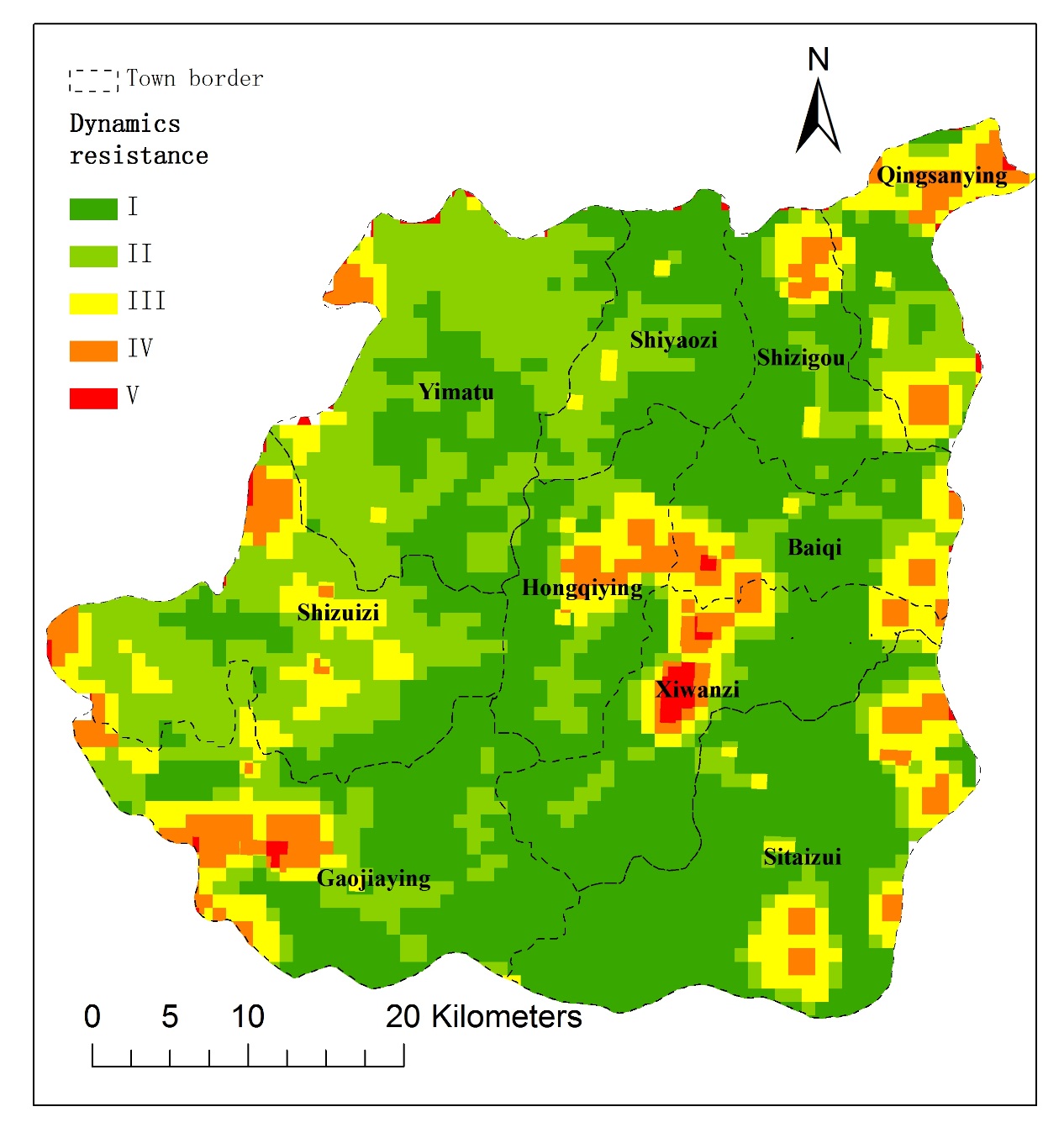


Figure S11 Spatial resistance patterns of ecological dynamics in Chongli (Generated by ArcGIS 10.1 software, http://www.esri.com).


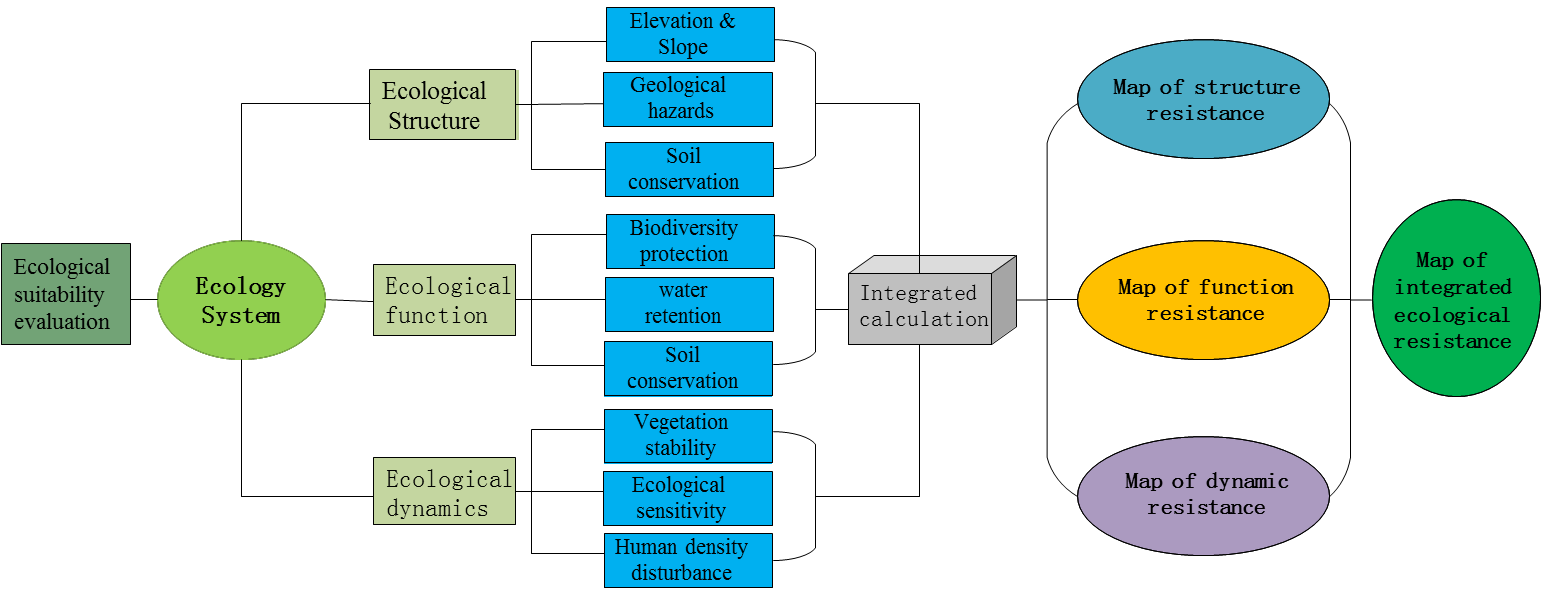


Figure S12 The process of integrated ecological resistances for construction in Yanghe Basin and Chongli district

Table S1 Spatial statistical analysis of ecological structure resistance in Yanghe Basin

| Area(km2) | V | IV | III | II | I | Total |
| --- | --- | --- | --- | --- | --- | --- |
| Chongli | 81.66 | 957.36 | 448.11 | 661.09 | 216.70 | 2364.92 |
| Huai'an | 24.05 | 267.56 | 72.43 | 511.26 | 812.88 | 1688.17 |
| Huailai | 338.46 | 437.80 | 91.50 | 278.86 | 648.22 | 1794.85 |
| Shangyi | 33.29 | 135.32 | 93.30 | 488.95 | 1908.84 | 2659.70 |
| Wanquan | 0.95 | 20.12 | 11.39 | 156.87 | 931.64 | 1120.97 |
| Xuanhua | 99.53 | 397.43 | 110.33 | 806.18 | 1100.32 | 2513.78 |
| Yangyuan | 106.62 | 249.40 | 77.47 | 248.28 | 1157.65 | 1839.41 |
| Zhangjiakou | 7.53 | 44.63 | 25.88 | 105.39 | 238.62 | 422.03 |
| Zhuolu | 673.41 | 756.07 | 142.74 | 588.46 | 611.32 | 2772.00 |
| Total | 1365.48 | 3265.70 | 1073.14 | 3845.33 | 7626.18 | 17175.83 |
| Proportion (%) | V | IV | III | II | I | Total |
| Chongli | 3.45 | 40.48 | 18.95 | 27.95 | 9.16 | 100.00 |
| Huai'an | 1.42 | 15.85 | 4.29 | 30.28 | 48.15 | 100.00 |
| Huailai | 18.86 | 24.39 | 5.10 | 15.54 | 36.12 | 100.00 |
| Shangyi | 1.25 | 5.09 | 3.51 | 18.38 | 71.77 | 100.00 |
| Wanquan | 0.08 | 1.80 | 1.02 | 13.99 | 83.11 | 100.00 |
| Xuanhua | 3.96 | 15.81 | 4.39 | 32.07 | 43.77 | 100.00 |
| Yangyuan | 5.80 | 13.56 | 4.21 | 13.50 | 62.94 | 100.00 |
| Zhangjiakou | 1.78 | 10.57 | 6.13 | 24.97 | 56.54 | 100.00 |
| Zhuolu | 24.29 | 27.28 | 5.15 | 21.23 | 22.05 | 100.00 |
| Total | 7.95 | 19.01 | 6.25 | 22.39 | 44.40 | 100.00 |

Table S2 Spatial statistical analysis of ecological function resistance in Yanghe Basin

| Area(km2) | V | IV | III | II | I | Total |
| --- | --- | --- | --- | --- | --- | --- |
| Chongli | 39.71 | 1340.78 | 846.82 | 124.27 | 10.02 | 2361.59 |
| Huai'an | 231.66 | 412.26 | 463.68 | 530.67 | 49.04 | 1687.32 |
| Huailai | 79.88 | 795.79 | 280.08 | 551.43 | 84.93 | 1792.11 |
| Shangyi | 205.91 | 549.20 | 1011.08 | 844.41 | 44.54 | 2655.15 |
| Wanquan | 42.92 | 129.79 | 498.53 | 402.74 | 46.32 | 1120.29 |
| Xuanhua | 86.01 | 799.32 | 866.67 | 683.24 | 77.66 | 2512.90 |
| Yangyuan | 134.56 | 374.75 | 358.24 | 889.05 | 77.84 | 1834.44 |
| Zhangjiakou | 28.70 | 87.22 | 98.28 | 109.70 | 98.14 | 422.05 |
| Zhuolu | 23.80 | 1688.09 | 527.65 | 479.79 | 51.43 | 2770.76 |
| Total | 873.16 | 6177.20 | 4951.04 | 4615.30 | 539.92 | 17156.61* |
| Proportion (%) | V | IV | III | II | I | Total |
| Chongli | 1.68 | 56.77 | 35.86 | 5.26 | 0.42 | 100.00 |
| Huai'an | 13.73 | 24.43 | 27.48 | 31.45 | 2.91 | 100.00 |
| Huailai | 4.46 | 44.41 | 15.63 | 30.77 | 4.74 | 100.00 |
| Shangyi | 7.76 | 20.68 | 38.08 | 31.80 | 1.68 | 100.00 |
| Wanquan | 3.83 | 11.59 | 44.50 | 35.95 | 4.13 | 100.00 |
| Xuanhua | 3.42 | 31.81 | 34.49 | 27.19 | 3.09 | 100.00 |
| Yangyuan | 7.34 | 20.43 | 19.53 | 48.46 | 4.24 | 100.00 |
| Zhangjiakou | 6.80 | 20.67 | 23.29 | 25.99 | 23.25 | 100.00 |
| Zhuolu | 0.86 | 60.93 | 19.04 | 17.32 | 1.86 | 100.00 |
| Total | 5.09 | 36.00 | 28.86 | 26.90 | 3.15 | 100.00 |

*Superposition analysis of different scales plaque will cause statistical error in the border areas

Table S3 Spatial statistical analysis of ecological dynamics resistance in Yanghe Basin

| Area(km2) | V | IV | III | II | I | Total |
| --- | --- | --- | --- | --- | --- | --- |
| Chongli | 49.64 | 124.23 | 232.78 | 706.54 | 1247.57 | 2360.77 |
| Huai'an | 39.14 | 151.73 | 816.23 | 559.67 | 117.62 | 1684.40 |
| Huailai | 251.02 | 208.81 | 244.31 | 305.73 | 782.55 | 1792.41 |
| Shangyi | 105.84 | 639.00 | 1609.34 | 273.06 | 25.29 | 2652.52 |
| Wanquan | 92.22 | 159.68 | 274.52 | 497.31 | 96.48 | 1120.22 |
| Xuanhua | 146.57 | 343.69 | 785.08 | 811.71 | 425.71 | 2512.76 |
| Yangyuan | 168.37 | 600.06 | 687.69 | 263.91 | 111.42 | 1831.45 |
| Zhangjiakou | 200.53 | 92.04 | 70.32 | 44.28 | 14.90 | 422.07 |
| Zhuolu | 91.96 | 295.99 | 360.97 | 493.97 | 1523.94 | 2766.84 |
| Total | 1145.29 | 2615.23 | 5081.24 | 3956.18 | 4345.49 | 17143.43 |
| Proportion (%) | V | IV | III | II | I | Total |
| Chongli | 2.10 | 5.26 | 9.86 | 29.93 | 52.85 | 100.00 |
| Huai'an | 2.32 | 9.01 | 48.46 | 33.23 | 6.98 | 100.00 |
| Huailai | 14.00 | 11.65 | 13.63 | 17.06 | 43.66 | 100.00 |
| Shangyi | 3.99 | 24.09 | 60.67 | 10.29 | 0.95 | 100.00 |
| Wanquan | 8.23 | 14.25 | 24.51 | 44.39 | 8.61 | 100.00 |
| Xuanhua | 5.83 | 13.68 | 31.24 | 32.30 | 16.94 | 100.00 |
| Yangyuan | 9.19 | 32.76 | 37.55 | 14.41 | 6.08 | 100.00 |
| Zhangjiakou | 47.51 | 21.81 | 16.66 | 10.49 | 3.53 | 100.00 |
| Zhuolu | 3.32 | 10.70 | 13.05 | 17.85 | 55.08 | 100.00 |
| Total | 6.68 | 15.26 | 29.64 | 23.08 | 25.35 | 100.00 |

*Superposition analysis of different scales plaque will cause statistical error in the border areas

Table S4 Spatial statistical analysis of ecological structure resistance in Chongli

| Area(km2) | V | IV | III | II | I | Total |
| --- | --- | --- | --- | --- | --- | --- |
| Baiqi | 10.12 | 24.23 | 35.11 | 38.09 | 48.27 | 155.82 |
| Gaojiaying | 26.21 | 49.92 | 30.36 | 127.00 | 111.43 | 344.92 |
| Hongqiying | 3.86 | 14.27 | 20.20 | 68.12 | 67.87 | 174.31 |
| Qingsanying | 6.08 | 11.50 | 15.40 | 58.80 | 49.08 | 140.86 |
| Shiyaozi | 3.19 | 12.49 | 25.89 | 48.75 | 55.59 | 145.91 |
| Shizigou | 6.95 | 15.68 | 28.80 | 36.55 | 38.29 | 126.28 |
| Shizuizi | 5.72 | 17.68 | 11.20 | 154.48 | 125.59 | 314.66 |
| Sitaizui | 47.18 | 66.78 | 87.14 | 71.38 | 103.67 | 376.14 |
| Xiwanzi | 24.80 | 42.13 | 44.09 | 58.63 | 61.55 | 231.20 |
| Yimatu | 6.99 | 24.28 | 25.80 | 155.08 | 137.81 | 349.96 |
| Total | 141.09 | 278.97 | 323.98 | 816.89 | 799.15 | 2360.08 |
| Proportion (%) | V | IV | III | II | I | Total |
| Baiqi | 6.50 | 15.55 | 22.53 | 24.45 | 30.98 | 100.00 |
| Gaojiaying | 7.60 | 14.47 | 8.80 | 36.82 | 32.31 | 100.00 |
| Hongqiying | 2.21 | 8.19 | 11.59 | 39.08 | 38.93 | 100.00 |
| Qingsanying | 4.32 | 8.16 | 10.94 | 41.74 | 34.84 | 100.00 |
| Shiyaozi | 2.18 | 8.56 | 17.74 | 33.41 | 38.10 | 100.00 |
| Shizigou | 5.50 | 12.42 | 22.81 | 28.94 | 30.33 | 100.00 |
| Shizuizi | 1.82 | 5.62 | 3.56 | 49.09 | 39.91 | 100.00 |
| Sitaizui | 12.54 | 17.75 | 23.17 | 18.98 | 27.56 | 100.00 |
| Xiwanzi | 10.72 | 18.22 | 19.07 | 25.36 | 26.62 | 100.00 |
| Yimatu | 2.00 | 6.94 | 7.37 | 44.31 | 39.38 | 100.00 |
| Total | 5.98 | 11.82 | 13.73 | 34.61 | 33.86 | 100.00 |

Table S5 Spatial statistical analysis of ecological function resistance in Chongli

| Area(km2) | I | II | III | IV | V | Total |
| --- | --- | --- | --- | --- | --- | --- |
| Baiqi | 3.80 | 5.98 | 58.54 | 85.68 | 2.57 | 156.57 |
| Gaojiaying | 8.69 | 13.18 | 117.16 | 189.81 | 16.16 | 345.01 |
| Hongqiying | 6.70 | 11.91 | 70.17 | 82.11 | 3.42 | 174.30 |
| Qingsanying | 13.04 | 19.79 | 77.87 | 29.01 | 2.52 | 142.23 |
| Shiyaozi | 10.34 | 25.47 | 82.61 | 25.66 | 2.15 | 146.23 |
| Shizigou | 8.50 | 16.09 | 68.22 | 32.13 | 1.46 | 126.41 |
| Shizuizi | 14.99 | 34.57 | 115.79 | 129.36 | 19.98 | 314.70 |
| Sitaizui | 9.04 | 13.02 | 81.04 | 262.64 | 11.48 | 377.22 |
| Xiwanzi | 4.71 | 6.24 | 41.65 | 174.35 | 4.29 | 231.23 |
| Yimatu | 22.99 | 51.86 | 125.45 | 141.07 | 9.08 | 350.45 |
| Total | 102.79 | 198.12 | 838.49 | 1151.84 | 73.11 | 2364.36 |
| Proportion (%) | I | II | III | IV | V | Total |
| Baiqi | 2.42 | 3.82 | 37.39 | 54.72 | 1.64 | 100.00 |
| Gaojiaying | 2.52 | 3.82 | 33.96 | 55.02 | 4.68 | 100.00 |
| Hongqiying | 3.84 | 6.83 | 40.25 | 47.11 | 1.96 | 100.00 |
| Qingsanying | 9.17 | 13.92 | 54.75 | 20.40 | 1.77 | 100.00 |
| Shiyaozi | 7.07 | 17.42 | 56.49 | 17.55 | 1.47 | 100.00 |
| Shizigou | 6.72 | 12.73 | 53.97 | 25.42 | 1.16 | 100.00 |
| Shizuizi | 4.76 | 10.98 | 36.80 | 41.11 | 6.35 | 100.00 |
| Sitaizui | 2.40 | 3.45 | 21.48 | 69.63 | 3.04 | 100.00 |
| Xiwanzi | 2.04 | 2.70 | 18.01 | 75.40 | 1.85 | 100.00 |
| Yimatu | 6.56 | 14.80 | 35.80 | 40.25 | 2.59 | 100.00 |
| Total | 4.35 | 8.38 | 35.46 | 48.72 | 3.09 | 100.00 |

Table S6 Spatial statistical analysis of ecological dynamics resistance in Chongli

| Area(km2) | I | II | III | IV | V | Total |
| --- | --- | --- | --- | --- | --- | --- |
| Baiqi | 91.97 | 23.53 | 26.73 | 13.19 | 0.97 | 156.41 |
| Gaojiaying | 197.05 | 82.89 | 45.96 | 16.66 | 2.08 | 344.65 |
| Hongqiying | 107.51 | 33.98 | 23.68 | 9.13 | 0.00 | 174.30 |
| Qingsanying | 46.50 | 45.58 | 38.27 | 10.43 | 1.26 | 142.04 |
| Shiyaozi | 96.27 | 45.47 | 3.98 | 0.00 | 0.36 | 146.08 |
| Shizigou | 86.50 | 21.86 | 13.71 | 3.99 | 0.19 | 126.25 |
| Shizuizi | 77.82 | 168.18 | 60.82 | 6.49 | 1.67 | 314.98 |
| Sitaizui | 274.97 | 37.25 | 44.48 | 19.68 | 0.20 | 376.59 |
| Xiwanzi | 159.89 | 25.24 | 25.30 | 13.62 | 7.18 | 231.24 |
| Yimatu | 109.60 | 222.23 | 13.17 | 1.89 | 1.70 | 348.59 |
| Total | 1248.09 | 706.21 | 296.11 | 95.10 | 15.63 | 2361.13 |
| Proportion (%) | I | II | III | IV | V | Total |
| Baiqi | 58.80 | 15.05 | 17.09 | 8.44 | 0.62 | 100.00 |
| Gaojiaying | 57.17 | 24.05 | 13.34 | 4.83 | 0.60 | 100.00 |
| Hongqiying | 61.68 | 19.50 | 13.59 | 5.24 | 0.00 | 100.00 |
| Qingsanying | 32.74 | 32.09 | 26.95 | 7.35 | 0.88 | 100.00 |
| Shiyaozi | 65.90 | 31.12 | 2.72 | 0.00 | 0.25 | 100.00 |
| Shizigou | 68.52 | 17.31 | 10.86 | 3.16 | 0.15 | 100.00 |
| Shizuizi | 24.71 | 53.39 | 19.31 | 2.06 | 0.53 | 100.00 |
| Sitaizui | 73.02 | 9.89 | 11.81 | 5.23 | 0.05 | 100.00 |
| Xiwanzi | 69.15 | 10.91 | 10.94 | 5.89 | 3.11 | 100.00 |
| Yimatu | 31.44 | 63.75 | 3.78 | 0.54 | 0.49 | 100.00 |
| Total | 52.86 | 29.91 | 12.54 | 4.03 | 0.66 | 100.00 |

Table S7 Ecological suitability assessment in Chongli

| Area(km2) | proir | Moderate | Potential | Restrict | Forbidden | Total |
| --- | --- | --- | --- | --- | --- | --- |
| Baiqi | 1.10 | 9.36 | 59.76 | 73.76 | 12.59 | 156.57 |
| Gaojiaying | 3.84 | 21.63 | 119.02 | 156.47 | 44.05 | 345.01 |
| Hongqiying | 1.36 | 14.14 | 71.70 | 77.60 | 9.50 | 174.30 |
| Qingsanying | 2.65 | 28.56 | 73.47 | 33.39 | 4.15 | 142.23 |
| Shiyaozi | 0.18 | 2.83 | 89.06 | 49.87 | 4.30 | 146.23 |
| Shizigou | 0.97 | 11.27 | 50.96 | 53.62 | 9.59 | 126.41 |
| Shizuizi | 2.07 | 32.21 | 179.61 | 92.41 | 8.41 | 314.70 |
| Sitaizui | 1.17 | 16.69 | 85.93 | 203.55 | 69.88 | 377.22 |
| Xiwanzi | 3.51 | 10.93 | 52.80 | 127.31 | 36.68 | 231.23 |
| Yimatu | 1.63 | 8.92 | 221.47 | 108.03 | 10.40 | 350.45 |
| Total | 18.48 | 156.54 | 1003.82 | 976.12 | 212.13 | 2367.10 |
| Proportion (%) | proir | Moderate | Potential | Restrict | Forbidden | Total |
| Baiqi | 0.71 | 5.98 | 38.17 | 47.11 | 8.04 | 100.00 |
| Gaojiaying | 1.11 | 6.27 | 34.50 | 45.35 | 12.77 | 100.00 |
| Hongqiying | 0.78 | 8.11 | 41.13 | 44.52 | 5.45 | 100.00 |
| Qingsanying | 1.87 | 20.08 | 51.66 | 23.48 | 2.92 | 100.00 |
| Shiyaozi | 0.12 | 1.94 | 60.90 | 34.10 | 2.94 | 100.00 |
| Shizigou | 0.77 | 8.92 | 40.31 | 42.41 | 7.59 | 100.00 |
| Shizuizi | 0.66 | 10.23 | 57.07 | 29.36 | 2.67 | 100.00 |
| Sitaizui | 0.31 | 4.42 | 22.78 | 53.96 | 18.53 | 100.00 |
| Xiwanzi | 1.52 | 4.73 | 22.83 | 55.06 | 15.86 | 100.00 |
| Yimatu | 0.47 | 2.54 | 63.20 | 30.83 | 2.97 | 100.00 |
| Total | 0.78 | 6.61 | 42.41 | 41.24 | 8.96 | 100.00 |

Table S8 Ecological suitability assessment in Yanghe Basin

| Area(km2) | proir | Moderate | Potential | Restrict | Forbidden | Total |
| --- | --- | --- | --- | --- | --- | --- |
| Chongli | 181.67 | 352.19 | 1276.00 | 549.52 | 7.02 | 2366.41 |
| Huai'an | 443.07 | 381.56 | 546.52 | 308.09 | 8.83 | 1688.07 |
| Huailai | 320.86 | 255.36 | 713.86 | 434.79 | 70.59 | 1795.45 |
| Shangyi | 572.02 | 709.24 | 1065.08 | 285.08 | 29.12 | 2660.54 |
| Wanquan | 419.28 | 344.22 | 316.56 | 40.72 | 0.35 | 1121.13 |
| Xuanhua | 520.07 | 615.36 | 1015.39 | 352.21 | 10.86 | 2513.89 |
| Yangyuan | 390.04 | 475.08 | 599.60 | 346.23 | 28.78 | 1839.74 |
| Zhangjiakou | 28.67 | 51.81 | 253.52 | 76.31 | 11.73 | 422.05 |
| Zhuolu | 398.52 | 332.91 | 1068.24 | 890.28 | 82.49 | 2772.44 |
| Total | 3274.21 | 3517.75 | 6854.77 | 3283.23 | 249.77 | 17179.72 |
| Proportion (%) | proir | Moderate | Potential | Restrict | Forbidden | Total |
| Chongli | 7.68 | 14.88 | 53.92 | 23.22 | 0.30 | 100.00 |
| Huai'an | 26.25 | 22.60 | 32.38 | 18.25 | 0.52 | 100.00 |
| Huailai | 17.87 | 14.22 | 39.76 | 24.22 | 3.93 | 100.00 |
| Shangyi | 21.50 | 26.66 | 40.03 | 10.72 | 1.09 | 100.00 |
| Wanquan | 37.40 | 30.70 | 28.24 | 3.63 | 0.03 | 100.00 |
| Xuanhua | 20.69 | 24.48 | 40.39 | 14.01 | 0.43 | 100.00 |
| Yangyuan | 21.20 | 25.82 | 32.59 | 18.82 | 1.56 | 100.00 |
| Zhangjiakou | 6.79 | 12.28 | 60.07 | 18.08 | 2.78 | 100.00 |
| Zhuolu | 14.37 | 12.01 | 38.53 | 32.11 | 2.98 | 100.00 |
| Total | 19.06 | 20.48 | 39.90 | 19.11 | 1.45 | 100.00 |

Table S9 Different types of ecosystems loss in the Genting Snow Part

| Area Function | Area name | Ecosystem loss area  （m^2^） | Area of ecosystem decreased（m^2^） | | | | |
| --- | --- | --- | --- | --- | --- | --- | --- |
|  |  |  | Coniferous Forest | Broad-leaved Forest | Shrub | Meadow | Barren land |
| Service security system area | Gondola | 17405.57 | 6548.86 | 7384.67 | 1032.64 | 2439.40 | 0 |
|  | Competition Management | 5638.8 | 94.736 | 221.04 | 174.24 | 3496.06 | 1652.73 |
| Service facilities construction area | Media | 46335.17 | 0 | 0 | 0 | 3480.86 | 1508.08 |
|  | Specting | 10389.31 | 2094.49 | 1178.15 | 613.26 | 4675.19 | 1828.23 |
|  | Venue Operation | 16300.61 | 3750.06 | 2715.55 | 3260.13 | 6574.87 | 0 |
| Main project Ski Area | Athlete | 45806.56 | 33235.99 | 30873.34 | 3733.52 | 5749.86 | 0 |
|  | Field of Play | 109376.93 | 24153.36 | 54598.03 | 8750.15 | 21875.39 | 0 |
|  | Trainning Course | 31375.9 | 20627.33 | 5144.45 | 1308.51 | 4295.62 | 0 |
| Infrastructure project area | Parking | 102088.2 | 5989.15 | 10344.96 | 0 | 44918.80 | 40835.28 |
|  | Road | 34590.9 | 1741.19 | 12759.42 | 323.30 | 12848.82 | 6918.17 |
|  | Olympic Family Hotel | 58651.83 | 0 | 0 | 0 | 18768.77 | 39883.06 |
| Environmental protection and management area | Genting snow park A/B surroding | 694738.22 | 153606.60 | 311867.96 | 55579.09 | 138947.65 | 34736.93 |
| Total |  | 1172698 | 251841.74 | 437087.58 | 74774.83 | 268071.28 | 123762.47 |

Table S10 The weight coefficient of judgment matrix of ecological suitability evaluation in Yanghe Basin

|  | Ecological  structure | Ecological  function | Ecological  dynamics | Data  sources |
| --- | --- | --- | --- | --- |
| Wi * | 1/3 | 1/3 | 1/3 | - |
| Slope** | 0.0535 |  |  | Generated by DEM using ARCGIS |
| DEM** | 0.4416 |  |  | Resources and Environment Science Data Center  http://www.resdc.cn |
| General geological  hazards** | 0.2137 |  |  | Zhangjiakou Municipal Bureau of land and resources |
| Earthquake** | 0.0937 |  |  | Zhangjiakou Municipal Bureau of land and resources |
| Distance from fragile area** | 0.1975 |  |  | Zhangjiakou Municipal Bureau of land and resources (data in 2013) |
| Biodiversity protection*** |  | 0.3333 |  | National earth system science data sharing infrastructure  <http://www.geodata.cn/> (data in 2015) |
| Water retention*** |  | 0.3333 |  | Resources and Environment Science Data Center  <http://www.resdc.cn> (data in 2015) |
| Soil conservation*** |  | 0.3333 |  | Using RUSLE model (data in 2013) |
| Vegetation stability**** |  |  | - | Maximus synthesis method to get NDVI value of each month from 2005~2015. Average value of monthly NDVI was calculated. Data was from 250m MODND1M  (MODIS, <http://www.gscloud.cn>, data from 2005 to 2015) |
| Ecological sensitivity**** |  |  | - | Zhangjiakou Municipal Bureau of land and resources (data in 2013) |
| Human density  disturbance**** |  |  | - | Resources and Environment Science Data Center (data in 2015) http://www.resdc.cn |

* Given that structures, functions and dynamics are interdependent based on the conceptual framework, three weights were given equal values. [^1^](#_ENREF_1)

**The weight of each index was calculated based on the Analytical Hierarchy Process (AHP). The results are very similar to another study of AHP weights in a mountainous region [^1^](#_ENREF_1). (The weights for slope, elevation, frequency of geological hazards, frequency of earthquakes, and distance to fracture zones are 0.0532, 0.4460, 0.2428, 0.1426, and 0.1154, respectively)

*** According to Peng’s study, ecological function could be calculated by an equally weighted sum of these 3 sub-indicators as the importance of biodiversity protection, water retention and soil conservation [^1^](#_ENREF_1)

**** Ecological dynamic value was calculated by Equation 2, according to vegetation stability, ecological sensitivity and human disturbance indicators. Ecologically sensitive areas include a collection of elements related to ecosystem elements that can cause changes in ecosystem structure and function and can cause a decline in ecosystem stability. We quantify the ecological sensitivity by using the distance between construction site and ecologically sensitive area. The ecologically sensitive areas of the Winter Olympics venue are mainly concentrated in nature reserves, such as 6 wetland type nature reserves, 4 forest and wild animal natural conservation areas, and 1 grassland type nature reserve.

| Weights | \|*S*\| | Index | \|*S*\| |
| --- | --- | --- | --- |
| Distance from fragile area | 0.357 | Distance from fragile area | 0.027 |
| DEM | 0.440 | DEM | 0.110 |
| Earthquake | 0.463 | Earthquake | 0.133 |
| Slope | 0.374 | Slope | 0.035 |
| geological hazards | 0.374 | geological hazards | 0.098 |
| Water retention | 0.455 | Water retention | 0.125 |
| Soil conservation | 0.496 | Soil conservation | 0.166 |
| Biodiversity protection | 0.331 | Biodiversity protection | 0.042 |
| Vegetation stability | - | Vegetation stability | 0.140 |
| Human density disturbance | - | Human density disturbance | 0.139 |
| Ecological sensitivity | - | Ecological sensitivity* | 0.140 |
| ws | 0.664 | - |  |
| wf | 0.735 | - |  |
| wd | 0.597 | - |  |

Table S11 The sensitivity analysis on the indexes and the weightings for one grid (N40.967°, E115.386°)

**Methods**

**Assessment of ecological structure**

Ecological structural resistance for land construction could be evaluated by ecological elements[^1^](#_ENREF_1) and be determined by elevation, slope and so on, which are shown in Table S10. The weight of each index was calculated by the method of Analytical Hierarchy Process (AHP) [^2^](#_ENREF_2)^,^[^3^](#_ENREF_3). The weights for each index are shown in Table S10. All the grid values were graded into five categories. The construction resistance caused by geological hazards and fracture zones can be classified on a descending scale from level 5 to level 1, with the levels corresponding to increasing distance to this region (0–200 m, 200–500 m, 500–1000 m, 1000–2000 m, and greater than 2000 m). DEM in Yanghe region were classified from level 1 to 5 with corresponding to the value of <500, 500-1000, 1000-1500, 1500-2000, >2000. Slope were classified from level 1 to 5, with corresponding to the value of <8°, 8-15°, 15-20°, 20-30°, >30°in Yanghe region. Similar classifications were shown in Peng’s studies[^1^](#_ENREF_1). Because DEM value in Chongli is relatively high, DEM classification were calculated by the method of natural breaks. In order to protect farmland in the flat areas, slope value 8-15°was defined as level 1 in Chongli. The evaluation of ecological structural resistance is the weighted sum of all the indices described above elements (Table S10).

**Assessment of ecological function**

In this study, spatial distribution of ecological function was evaluated by three vital ecological importance, biodiversity protection, water retention, and soil conservation. The biodiversity protection importance in different ecosystems is assessed based on the average value of ecosystem services of landscape plaque to maintain biodiversity in China[^1^](#_ENREF_1)^,^[^4^](#_ENREF_4)^,^[^5^](#_ENREF_5). Biodiversity protection is linked with ecosystem types as follows: level1 refers to construction land and unused land, level 2 farmland, level 3 gardens and lawns, level 4 is woodland, and level 5 surface water and wetland. Water retention classification could be assessed according to vegetation type[^1^](#_ENREF_1). The level for water retention capacity is linked with vegetation types as follows: non-forested land as level 1; agricultural land, grazing land and nursery as level 2; shrub land, economic forests and grass land as level 3; broadleaf forests and thin forests as level 4; coniferous forests, wetlands and water bodies as level 5. Soil conservation was evaluated by the revised universal soil loss equation (RUSLE, equation 1), and the factors of this equation was collected in Li’s study[^6^](#_ENREF_6).

$A= R\times K\times LS \times C\times P$ (1)

*A*= average annual soil loss

*R*= rainfall-runoff erosivity factor

*K*= soil erodibility factor

*LS*= slope length and steepness factor

*C*= cover management factor

*P*= support practice factor

**Assessment of ecological dynamics**

Ecological dynamics assessment was provided by Peng’s concept method[^1^](#_ENREF_1). It reflect the temporal dynamic characteristics of a natural ecosystem’s self-organization and self-update. Vegetation stability (S1), ecological sensitivity (S2), and human disturbance (S3) was regards as importance indicators to depict aspects of resistance, exposure, and interference of ecological dynamics. Ecological dynamic resistance *ER* can be expressed as:

$ER=\frac{S1\times S2}{S3}$ (2)

where S1 is quantified by NDVI value; S2 is the reciprocal of distance to the nearest key ecological sensitivity areas; S3 refers to the spatial differentiation of human activity, expressed by spatial population density.

**References**

1 Peng, J., Ma, J., Du, Y., Zhang, L. & Hu, X. Ecological suitability evaluation for mountainous area development based on conceptual model of landscape structure, function, and dynamics. *Ecological Indicators* **61**, 500-511 (2016).

2 Javadian, M., Shamskooshki, H. & Momeni, M. Application of Sustainable Urban Development in Environmental Suitability Analysis of Educational Land Use by Using Ahp and Gis in Tehran. *Procedia Engineering* **21**, 72-80 (2011).

3 Yu, J., Chen, Y., Wu, J. & Khan, S. Cellular automata-based spatial multi-criteria land suitability simulation for irrigated agriculture. *International Journal of Geographical Information Science* **25**, 131-148 (2011).

4 Shi, T., Zheng, G., Wang, Z. & Wang, L. Progress in Research on Land Suitability Evaluation in China. *Progress in Geography* **26**, 106-115 (2007).

5 Xie, G. D., Zhen, L., Lu, C.X., Xiao, X., Chen, C.,. Expert knowledge based valua-tion method of ecosystem services in China. . *J. Nat. Resour.* **23**, 911-919 (in Chinese) ( 2008).

6 Li, F. *Study on Soil Erosion in Hebei Province Based on USLE Model* Master thesis, Hebei Normal University, (2013).
